# Killing in the name of: T6SS structure and effector diversity

**DOI:** 10.1099/mic.0.001367

**Published:** 2023-07-25

**Authors:** Luke P. Allsopp, Patricia Bernal

**Affiliations:** ^1^​ National Heart and Lung Institute, Imperial College London, London, UK; ^2^​ Departamento de Microbiología, Facultad de Biología, Universidad de Sevilla, Sevilla 41012, Spain

**Keywords:** Type VI secretion system, T6SS, antimicrobial effectors, antieukaryotic effectors, T6SS assembly, membrane complex, baseplate, tail, inner tube, contractile sheath

## Abstract

The life of bacteria is challenging, to endure bacteria employ a range of mechanisms to optimize their environment, including deploying the type VI secretion system (T6SS). Acting as a bacterial crossbow, this system delivers effectors responsible for subverting host cells, killing competitors and facilitating general secretion to access common goods. Due to its importance, this lethal machine has been evolutionarily maintained, disseminated and specialized to fulfil these vital functions. In fact, T6SS structural clusters are present in over 25 % of Gram-negative bacteria, varying in number from one to six different genetic clusters per organism. Since its discovery in 2006, research on the T6SS has rapidly progressed, yielding remarkable breakthroughs. The identification and characterization of novel components of the T6SS, combined with biochemical and structural studies, have revealed fascinating mechanisms governing its assembly, loading, firing and disassembly processes. Recent findings have also demonstrated the efficacy of this system against fungal and Gram-positive cells, expanding its scope. Ongoing research continues to uncover an extensive and expanding repertoire of T6SS effectors, the genuine mediators of T6SS function. These studies are shedding light on new aspects of the biology of prokaryotic and eukaryotic organisms. This review provides a comprehensive overview of the T6SS, highlighting recent discoveries of its structure and the diversity of its effectors. Additionally, it injects a personal perspective on avenues for future research, aiming to deepen our understanding of this combative system.

## Introduction

**Fig. 1.**
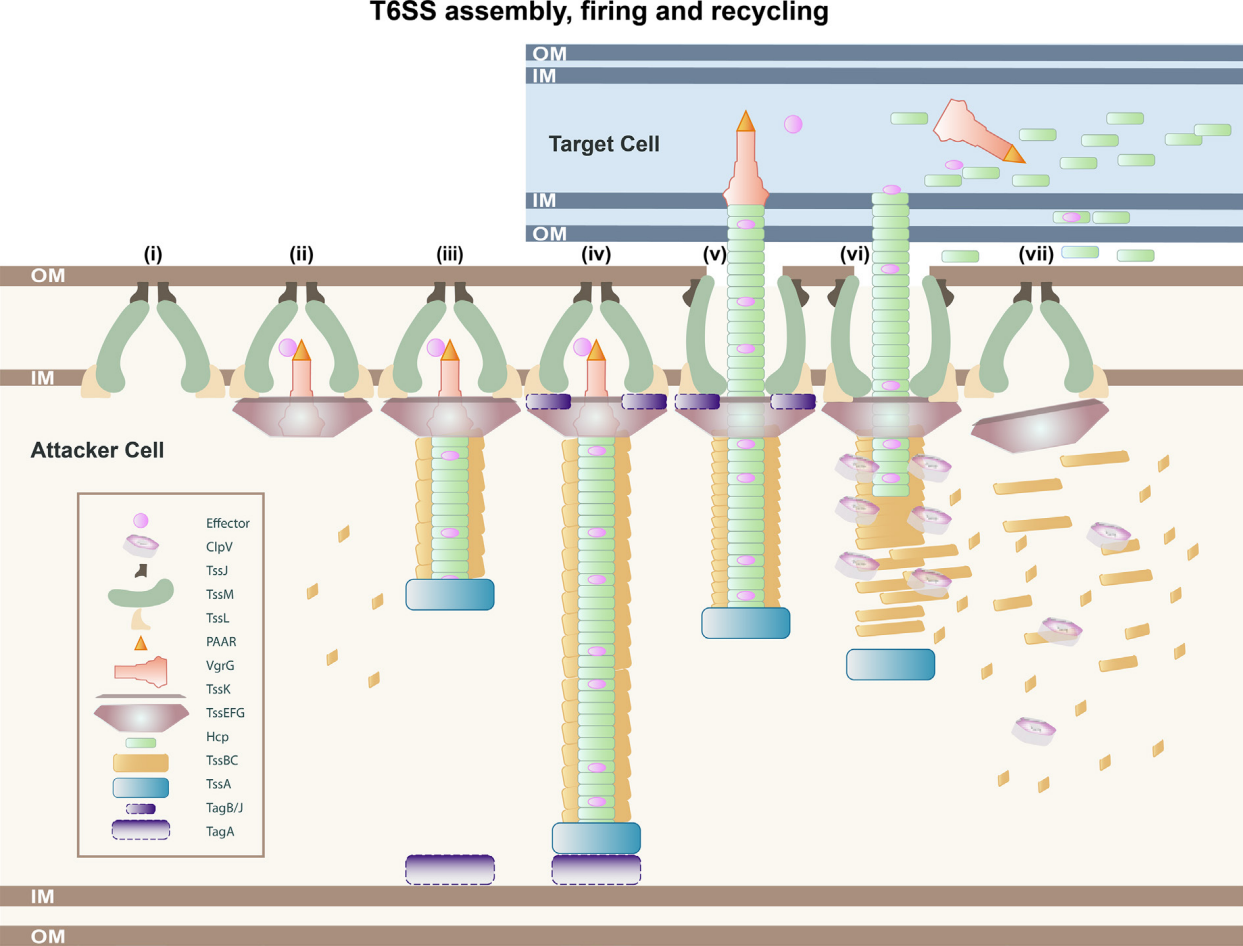
Schematic representation of a T6SS being assembled, fired and recycled. (i) The membrane complex (TssJLM) inserts and assembles in the membrane. This tethers the system to the cell envelope; (ii) and recruits the baseplate (TssEFGK) structure that is shown in brown. The VgrG/PAAR spike complex (red/orange) is the hub of the baseplate. Effectors (pink) can be loaded to the spike complex. (iii) TssA protein (blue) primes polymerization of the tail from the VgrG hub and is located at the end of the growing structure. The tail is composed of the inner Hcp tube (green) with loaded effectors (pink) and the outer contractile TssBC sheath (yellow). (iv) The tail polymerizes until it reaches the opposite side of the cell. Depending on the systems, the sheath can be stabilized by TagA or TagB/J accessory proteins (purple). TagA is recruited by TssA to clamp the end of the sheath to the opposing cell membrane alternatively TagB/J are recruited to the baseplate by TssA. (v) Upon a signal, a conformational change in the baseplate triggers the progressive contraction of the sheath from the baseplate that propels the inner tube, spike and the associated effectors outside and into the target cell (or environment). (vi) The contracted sheath is disassembled for recycling by the ATPase ClpV. (vii) The membrane complex remains and it can be reused to assemble a new T6SS. Model depicts a Gram-negative bacterial prey cell, but this could also be a eukaryotic cell or as recently shown a fungal cell or Gram-positive bacterium. (Inset) A key to the colours and shapes used to depict T6SS protein components.

The type VI secretion system (T6SS) is a dynamic bacterial nanomachine that allows micro-organisms to deliver effector proteins extracellularly or directly into target cells, including both prokaryotes and eukaryotes (Fig. 1). The first identified T6SS effectors were antieukaryotic and disrupted the physiology of the target host cell [1]. However, a series of breakthroughs and subsequent developments in the field of T6SS have shown that most T6SS effectors have antibacterial activity. Recent research has broadened this view by showing that some effectors encode antifungal activities, which expands the defined functions of T6SS from an antibacterial to an antimicrobial machine [2]. The demonstration that the T6SS also acts in general secretion with the delivery of effector proteins into the extracellular environment to access common goods has further shown its utility [3, 4]. Advances in the characterization of T6SS effectors have revealed a wide variety of mechanisms that are used to inhibit prey cells, including membrane damage by the formation of pores or direct disruption, inhibition of cell-wall synthesis, degradation of nucleic acids, or inhibition of target processes such as protein biosynthesis [5, 6]. During infection, the T6SS effectors exert control over the eukaryotic host in various manners, including polymerising its cytoskeleton, dampening the immune response, or altering the signalling of host cells [7, 8]. The effectors secreted into the extracellular environment play a vital role in bacterial survival by capturing essential elements such as iron, magnesium or manganese, which are present in limited concentrations in ecological niches and at the same time reducing their availability to competitors resulting in their growth restriction [3]. Thus, by delivering T6SS effectors, these nanomachines provide multiple benefits to the bacteria that possess them, including increased competitiveness in mixed microbial environments, improved colonization of host organisms, modulation of host immune responses, increased resistance to predation and environmental stressors, and greater potential for adaptation and evolution. Genomic analysis has shown that T6SS clusters are present in over 25 % of Gram-negative bacteria with individual strains encoding between 1 and 6 distinct T6SS clusters [9, 10]. This widespread distribution suggests that their usefulness has conferred an evolutionary advantage, leading to their selection, and that bacteria have developed regulatory mechanisms to minimize associated costs or for deployment in appropriate circumstances for bacterial benefit.

**Fig. 2.**
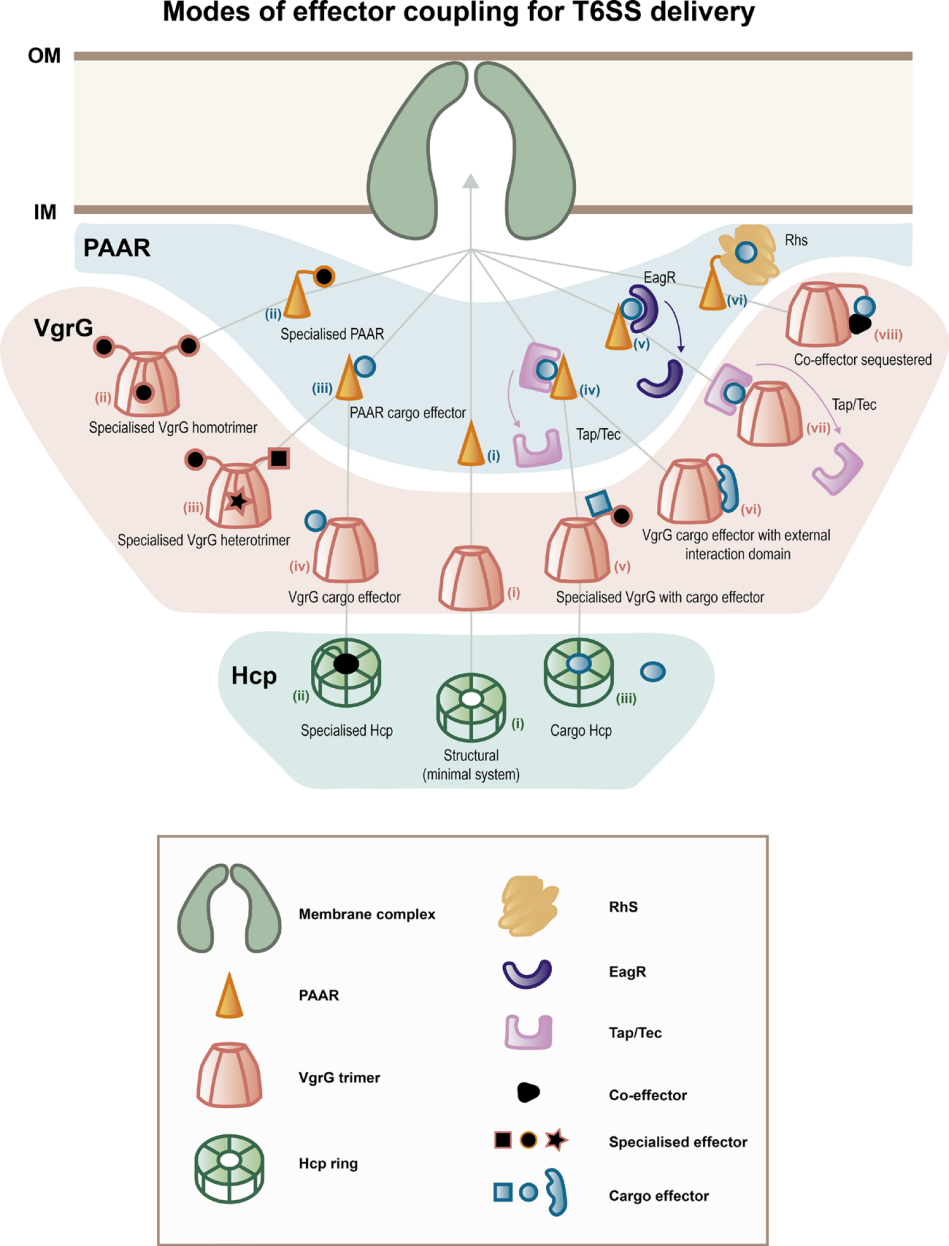
Modes of effector coupling to T6SS components for T6SS delivery. Model representing the variability encountered during assembly of the structural components of the T6SS that are secreted, i.e. the spike complex (PAAR and VgrG trimer) and the Hcp inner tube; both of which can be loaded with effectors. Exclusively one PAAR protein (shaded blue) is loaded per spike onto a trimer of VgrG proteins (shaded red) and followed by the addition of the Hcp rings (shaded green). Grey arrows represent step-wise binding and loading and are not restrictive paths, i.e. multiple potential combinations of the components can assemble a loaded system. (Note that different combinations of assemblies can also occur, e.g. different PAARs with different VgrGs or specialized VgrGs with VgrGs that load cargo effectors in the one trimer that are not depicted for simplicity). Pink/purple arrows depict components (chaperones/adaptors) that are required for loading effectors onto the system (Eag/Tec/Tap) (Note that Tec/Tap proteins can have additional interaction partners named co-Tec proteins that are not shown). The secretion of these proteins has not been demonstrated so are suggested to be stripped off during effector loading. Hcp proteins can either be (**i**) structural with no effector loaded, (ii) specialized (an extra effector domain of a Hcp protein) or (iii) Hcp acting as a chaperone for loading of a cargo effector. Models of VgrG trimers: (i) simplest configuration, with purely structural proteins depicted and no effectors loaded, (ii) specialized VgrG, where additional effector domains are present as extensions of the VgrG protein. Depending on the number of VgrGs secreted by a single system, they can have homotrimers or (iii) specialised VgrG heterotrimer with different effectors loaded. It can also have specialised VgrGs trimerising with purely structural VgrGs or VgrGs that bind cargo effectors (not shown). (iv) VgrG with cargo effector bound for secretion/delivery (can also have three VgrGs with each interacting with a cargo effector). (v) Specialized VgrG can encode additional domains that facilitate loading of a cargo effector. (vi) VgrG with cargo effector that has external interaction domains. (vii) VgrG with chaperone/adaptor assisted loading of an effector with a Tec/Tap protein. No evidence of Tec/Tap secretion. (viii) VgrG with co-effector. Effectors and co-effectors are required for binding to a VgrG and are both secreted. Models of PAAR proteins: (**i**) simplest configuration, with only a structural role for hardening of the T6SS spike complex and no effectors loaded. (ii) Specialized PAAR with effector domain encoded in the one protein. (iii) PAAR with cargo effector loaded. (iv) PAAR with cargo effector loaded that requires a Tap/Tec adaptor/chaperone protein for effector coupling. No evidence for Tap/Tec secretion. (v) PAAR with cargo effector loaded that requires a Eag adaptor/chaperone protein. No evidence for Eag secretion. (vi) PAAR with Rhs effector protein loaded. (Note that Rhs effectors can be specialized or cargo effectors and may require a chaperone/adaptor protein or domain that is not shown in this model for simplicity). Different combinations of PAAR, VgrG and Hcp proteins can result in a payload of effector proteins to the extracellular environment or directly into target cells. These effectors can have synergistic functions, but a combination of effectors also increases the chances that T6SS puncture will deliver effectors that are functional in different targets.

In the last decade, significant progress on the structure of the T6SS has improved our understanding of the roles of individual components and their interactions during assembly, as well as identifying key regulatory factors that control the assembly process [11]. This knowledge has led to a solid understanding of the well-conserved machinery at the molecular level and the stepwise progression of the T6SS assembly and firing dynamics. At the structural level, the T6SS can be defined as a contractile nanomachine that spans the bacterial envelope. It consists of a membrane complex anchored to the cell wall that docks a baseplate, which facilitates the extension of a contractile sheath that wraps around a tube caped by the spike (Fig. 1). The inner tube and the spike are loaded with the above-mentioned effector proteins that are delivered to the target cell upon contraction of the sheath (Figs 1 and 2). Four different subtypes of T6SSs have been described in the literature (T6SS^i-iv^), with variations in the number of conserved components, low homology among subtypes proteins and nuances in their structural assemblies. Here, we focus on the structure and mechanism of action of the canonical T6SS^i^, found mainly in Proteobacteria [1, 12]. The T6SS^i^ can be divided into six subfamilies named 1, 2, 3, 4a, 4b and 5 based on phylogenetic analysis and the conservation of accessory proteins [10, 13, 14]. We also mention subtypes T6SS^ii^ and T6SS^iii^ that exist exclusively in the *Francisella* genus and the *Bacteroidetes* phylum, respectively [15–17]. The T6SS^iv^, which is observed only in *Amoebophilus* [18] is not discussed in this review. This system lacks the proteins of the cell envelope (TssJML) and the recycling component (ClpV). T6SS^iv^ forms hexagonal arrays of T6SSs to coordinate firing events but maintain a similar firing mechanism of T6SS^i^ [18]. Thus, despite these variations many T6SS concepts are broadly applicable.

**Fig. 3.**
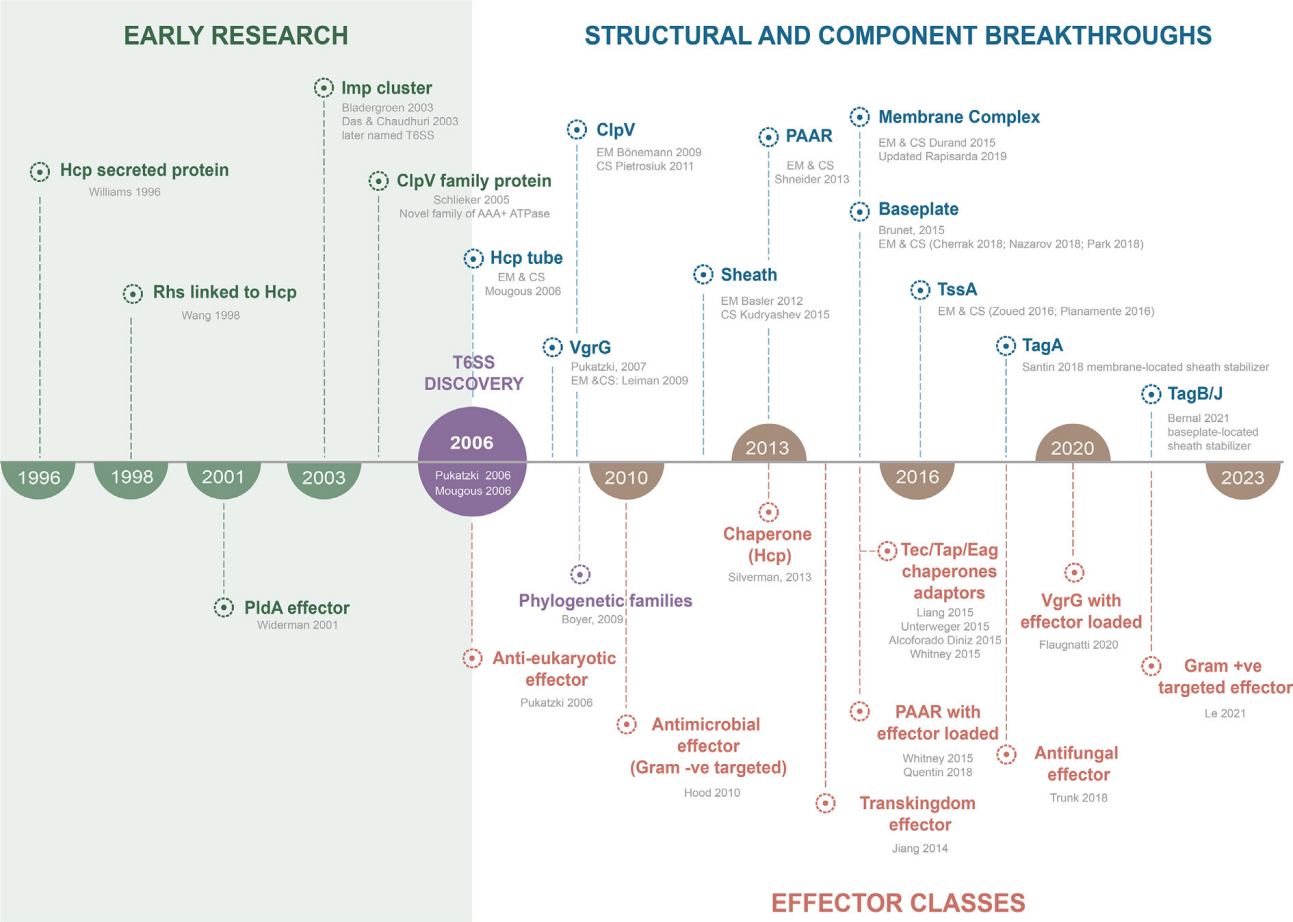
Timeline infographic of T6SS research covering early studies before the T6SS was coined to recent advancements. Graphic highlights key structural breakthroughs and milestones including when the effector classes were first identified in the literature. EM=electron microscopy, CS=crystal structure.

In this review, we compile a historical view of the evolution of the T6SS field over the last two decades (Fig. 3), focusing on the structural architecture of this fascinating nanomachine and highlighting developments on the diverse activities of T6SS effectors. Among the great variety of effector proteins, we selectively highlighted a subset to provide illustrative examples of different types and activities. We present here an engaging perspective on these two foundational pillars, structure and effectors, of the T6SS discipline by not only reviewing the seminal works that have shaped the field, but also exploring new avenues that could facilitate the expansion of the field.

## The mechanics of the T6SS

Construction of the T6SS nanomachine is a multistep process that requires the coordinated action of numerous proteins for sequential incorporation and assembly. In essence, it involves the assembly of a membrane-bound platform, docked to a baseplate and a puncturing device wrapped in a contractile sheath. Once fired, the device delivers a payload of effector proteins. This action is followed by the disassembly and recycling of the T6SS components (Fig. 1).

The T6SS architecture exhibits evolutionary, structural and functional parallels with the tails of contractile bacteriophages. The structure of the system can be divided into three main sections: the membrane, the baseplate and the tail complexes. Whilst the baseplate and tail subcomplexes of T6SS are related to phages [19], the membrane complex presents clear homology with the IcmF and DotU proteins of the type IVb secretion system (T4bSS) [20]. These complexes have been evolutionarily and structurally co-opted to facilitate a phage-like firing mechanism to be secured to the Gram-negative cell envelope and fire out of bacterial cells. This process enabled the genesis of a new secretion system apparatus known as the T6SS.

Assembly begins with the insertion and anchoring of the membrane complex, composed of TssJML, to the cell envelope (Fig. 1i) [21]. This structure facilitates the cytoplasmic docking of the baseplate complex, composed of TssKEFG, at the inner membrane. A trimer of valine-glycine repeat G (VgrG) proteins capped by a proline-alanine-alanine-arginine) PAAR domain forms the spike of the system and also acts as a hub for baseplate assembly (Fig. 1ii) [22]. The tail-like structure is assembled from the baseplate directed by the T6SS coordinator, the TssA protein (Fig. 1iii) [23]. Polymerization of the two structures of the tail, the inner tube (hemolysin co-regulated protein, Hcp) and the contractile sheath (TssB/C), takes place coordinately and ends when it reaches the other side of the cell (Fig. 1iv) [24]. Upon receiving a signal from the membrane complex, the baseplate is thought to undergo a conformational change that triggers a rapid sequential contraction of the sheath. This action drives the inner tube capped by the spike through the system and out of the cell to deliver the effectors (Fig. 1v). Then, the ATPase unfoldase ClpV disassembles the contracted sheath to recycle its components for additional rounds of firing [25] (Fig. 1vi–vii).

In this section, we sequentially illustrate the structural features of the system, the molecular mechanism that underlies the assembly of the T6SS components, and the regulatory factors that can control this process at the protein level.

## The membrane complex

### Docking the system to the cell envelope

The membrane complex (MC) is required for the nucleation of the T6SS assembly in a specific membrane location and defines the orientation of the tail. MC formation begins with TssJ, a periplasmic lipoprotein attached to the outer membrane (OM) by an acyl anchor [26]. Subsequently, the carboxy-terminal domain of the inner membrane (IM) protein TssM interacts with TssJ in the periplasm and OM [27], allowing the formation of transient pores in the OM (Fig. 1i) [28]. The IM protein TssL is the last component incorporated into the MC and interacts with TssM in the IM and the cytoplasmic space [21, 28]. The best studied MC is that of Enteroaggregative *Escherichia coli* (EAEC) that has a 4.9 Å cryo-electron microscopy structure with a fivefold symmetry, a TssJ:TssM:TssL 3 : 2 : 2 stoichiometry and a size of approximately 1.7 megadalton [21]. The assembled complex encompasses a large base (10 copies of TssL and TssM cytoplasmic domains) in the inner membrane-cytoplasm interphase and a double-ring shape (15 copies of TssJ and 10 copies of TssM periplasmic domains) in the periplasm, which collectively forms a bell-shaped transmembrane spanning structure (Fig. 1i) [21, 28]. This structure houses the spike and dynamically opens to allow the passage of the tail inner tube and the puncturing device through the Gram-negative cell envelope (Fig. 1ii–vi) [21, 28]. After firing, the MC remains in place and is reused by the cell to reassemble new T6 machines at the same location (Fig. 1i–vii). This concept is supported by the observation of sheath re-assembly (elongation) at the original position after sheath disassembly that follows a firing event [28–30].

### Variations and auxiliary proteins of the MC

In addition to the three-core membrane complex forming proteins (TssJLM), several T6SS gene clusters encode membrane proteins containing peptidoglycan-binding (PGB) domains. In some cases, the PGB domain is fused to the C-terminal domain of the core IM component TssL, which is then termed an evolved TssL, similar to that found in *Salmonella enterica* T6SS or TssL1 from *Pseudomonas aeruginosa* H1-T6SS [31]. In other cases, the PGB domain is an additional protein that is classified as an accessory component, for example, the type VI secretion associated gene L, TagL, which is an inner membrane PGB domain-containing protein found in EAEC that is recruited to the membrane complex by TssL [32, 33]. In *Pseudomonas putida *, a TssM derivative carrying a PGB instead of the periplasmic C-terminal domain has been identified *in silico* within the K1-T6SS cluster and named TagP [31, 34]. Other genes near T6SS clusters have been identified to encode predicted PGB domains. Among these are TagN and TagW, whose locations and roles in T6SS assembly remain to be characterized [31]. Proteins with PGB motifs allow the T6SS machinery to attach to the PG and thus to the cell wall. These proteins are not T6SS core components and do not seem to be necessary to assemble the MC [33]. However, some T6SS machines require PGB domain-containing proteins for proper function, such as EAEC T6SS [31, 33]. Recently, a novel accessory component of the T6SS with a PGB domain (SH3b) has been described in *Serratia marcescens* and named TagV. This protein is an outer membrane lipoprotein like TssJ but is proposed to bind peptidoglycan. Curiously, TagV does not bind to any other MC component and is likely not part of the MC itself. It is hypothesized that TagV could enhance PG rearrangement before the MC assembles or it may stabilize the complex. In this system, neither of the outer membrane lipoproteins is completely essential, but the presence of both proteins (TssJ and TagV) is necessary for full T6SS activity [35].

The TssJLM complex is approximately 18 nm wide in the periplasm, exceeding the size of the peptidoglycan pores (⁓2 nm). This raises the question of how the T6SS membrane complex passes through the peptidoglycan layer. Enzymes that hydrolyse PG for the proper insertion of the MC into the cell wall would be the solution, but genes encoding this type of enzymes are not commonly encoded within the T6SS genetic clusters [10]. Curiously, a gene conserved in the T6SS cluster of several bacterial species that encodes an endopeptidase was identified within the T6SS cluster of *Acinetobacter baumannii* in 2016. This endopeptidase, named TagX, has been proposed to regulate the digestion of peptidoglycan to allow the insertion and assembly of the T6SS machinery [36]. Soon after, a housekeeping lytic transglycosylase (LTG), MltE, was shown to be essential for T6SS assembly in EAEC. This enzyme is recruited to the MC as it assembles in the periplasm. The activity of MltE is specifically stimulated by the C-terminal domain of TssM, allowing the spatial regulation of the enzyme activity in proximity to the assembly point. The hydrolytic activity of MltE seems to be required for the polymerization of the TssJLM heterotrimer and thus for the final assembly of the MC [37]. Specialized LTGs are commonly found encoded in genetic clusters of structures that span the cell envelope, such as T3SS, T4SS, flagella, and type IV pili [38] but are an exception in T6SS clusters. As a result, the assembly of the T6SS membrane complex most likely requires the domestication of a housekeeping LTG from the T6SS-producing bacteria to reorganize the cell wall for insertion.

### TssA, the integrator

The MC serves as a connector to dock the baseplate assembly platform to the cell envelope and form a channel for the inner tube and the spike to cross the cell envelope upon sheath contraction (Fig. 1). The protein responsible for the connection between these T6SS subcomplexes and therefore crucial for the biogenesis of T6SS is TssA. TssA forms a dodecameric complex that binds to the TssJM complex before TssL is recruited to the MC. Then, TssA recruits the baseplate platform and initiates the polymerization of the tail (Fig. 1iii). Once the baseplate is recruited to the MC, TssA is displaced to the distal end of the baseplate to prime the polymerization of the tail (Fig. 1iii-iv) [23]. TssA is considered a core structural component essential for T6SS function; however, despite this, it is not a well-conserved protein among the different T6SS phylogenetic groups. The TssA proteins exist in two major forms: a short 40 kDa version (TssA_S_) and a longer 60 kDa protein (TssA_L_) [30]. Although both have a conserved N-terminal domain known as ImpA, only TssA_L_ harbours a C-terminal VasJ domain [23, 39]. The TssA_L_ proteins present in T6SS machines from *Escherichia coli *, *Vibrio cholerae* and *Aeromonas hydrophila* form a ringlike structure, with the C-termini occupying most of the centre of the ring [23, 40, 41]. These long TssA proteins interact with an accessory protein, i.e. TagA, that anchors the distal end of the sheath to the opposing side of the cell membrane (Fig. 1iv). This enables optimal polymerization of the sheath and allows this structure to remain primed for firing in an extended state for long periods of time [41, 42]. On the other hand, short TssA proteins including those of *P. putida* K1-T6SS and *P. aeruginosa* H1-T6SS, interact with different accessory proteins, i.e. TagB/TagJ, that are recruited to the baseplate during tail polymerization (Fig. 1iv). TagB/TagJ stabilizes the polymerizing sheath in the extended state to ensure the complete assembly of T6SS across the entire length of the cell followed by immediate contraction once this is achieved [30]. In conclusion, the two forms of TssA proteins are critical and play similar but distinct roles in keeping the T6SS sheath in a stable conformation until assembly is completed for efficient firing.

### The baseplate: the tail polymerization platform

The baseplate (BP) is composed of the highly conserved protein complex TssEFGK and the VgrG-PAAR subunits that will form the spike of the system and upon which polymerization of the tail occurs [43]. The BP also serves as a station for attaching and arranging the spike-attached effectors prior to delivery (Fig. 1ii). Cryo-electron microscopy reconstructions of several model organisms have revealed that two TssK trimers interact with a complex comprising two subunits of TssF and one subunit of TssG together with TssE to form a wedge. A circular array of six of these wedges constitutes the basic baseplate structure that encompasses 36 subunits of TssK, 12 subunits of TssF and six subunits of both TssG and TssE [22, 44, 45]. The wedge complexes assemble around the central hub of the spike complex, that is composed of a VgrG trimer capped by a PAAR-repeat protein, to form the entire baseplate.

The TssK trimers in the BP complex enable the recruitment of the BP to the MC through interaction with the cytoplasmic domain of TssL. Interestingly, the membrane complex and the baseplate have different evolutionary origins coming from the T4bSS [20] and the T4 phage [19], respectively. Thus, a key step in the genesis of the T6SS gene cluster must have been the recombination and specialization of genes from the two systems mentioned above that resulted in the codification of new proteins that enabled T6SS docking. As expected, these encoding genes (*tssK-tssL*) are now genetically linked in nearly all T6SS clusters [10] reinforcing the concept that they have co-evolved [46]. At this interface, the N-terminal shoulder domain of TssK binds to the baseplate components TssFG and the C-terminal head domain mediates interactions with the cytosolic domains of the TssL inner membrane protein [46–49]. The TssKFG complexes polymerize around the VgrG hub even in the absence of TssE. TssE connects to the BP at a later stage and has been suggested to initiate the polymerization of the contractile sheath [22, 45]. Direct interactions between the BP components TssE and TssG and the cytosolic domain of TssL and TssM have also been identified [43, 50]. These additional contacts, which are not present during BP biogenesis, could be the result of structural rearrangements that stabilize the interactions between the MC and the BP after sheath contraction (Fig. 1v) [22].

Once anchored, the BP complex acts as the assembly platform for the tail tube/sheath subcomplex with BP components interacting with the inner tube (Hcp hexamers) and the contractile sheath (TssBC) (Fig. 1iii). The model by [22] suggested that TssF interacts with the N-terminal domains of TssBC and that TssFG interacts with Hcp [43]. At the same time, TssG and TssE could stabilize the BP-sheath interface by interacting with the C-terminal domain of TssB [51]. TssE is crucial for sheath elongation and stability in *P. aeruginosa* [49] and is the sheath initiator in *V. cholerae* [45]. Once the BP is coupled to the MC and the interactions between the BP components and the Hcp and TssB/C proteins begin to occur, the next stage of assembly takes place.

### Post-translational regulation of the T6SSs

The assembly of the T6SS at these early stages of MC and BP formation can be regulated by several post-translational mechanisms that vary depending on the bacterium. The most well-studied are the PpkA/PppA serine–threonine kinase/phosphatase system and TagF and Forkhead-associated (FHA) domain-containing proteins [52, 53]. These systems are interconnected and can block the assembly of the T6 machinery in the early stages. Interactions between the MC components can be regulated by their phosphorylation (*p*-) state or by the binding or release of the regulatory proteins. For instance, in *P. aeruginosa *, PpkA phosphorylates (*p*-) Fha1 (*p*-Fha1), which is required for the assembly of H1-T6SS [52]. PppA removes the phosphorylation of Fha1 functioning as a post-transcriptional repressor. Proteins encoded by the T6SS associated genes *tagQRST* form a complex in the bacterial envelope and form a signal-transmission module that responds to membrane perturbations, such as antibiotics or T6SS attack, that ultimately phosphorylates Fha1 [54–57] and promotes H1-T6SS assembly. In this system, TagQ and TagR are the sensors that promote PpkA phosphorylation and control the post-translational assembly of the baseplate component TssK, presumably through Fha1 phosphorylation [49].

As the formation of TssK complexes is essential for the baseplate to form and allows the recruitment and assembly of the rest of the contractile apparatus, this acts as a central control point for T6SS functionality [49]. PppA phosphatase activity is important for TssK disassembly and repositioning of the baseplate to a membrane complex in new positions in the cell [49]. In contrast, in *Agrobacterium tumefaciens,* PpkA directly phosphorylates the membrane complex component TssL [58]. This has been proposed to trigger a conformational shift in TssM and enable Fha to interact with *p*-TssL to allow tail subassembly resulting in a functional T6SS [58]. A phosphorylation-independent pathway, usually governed by TagF, acts as a post-translational repressor of the systems and the encoding genes are observed in many T6SS clusters. In *P. aeruginosa *, *A. tumefaciens* and *S. marcescens *, TagF interacts with Fha and negatively impacts T6SS assembly at the connection between the MC and the BP [53, 54, 59]. Interestingly, in *S. marcescens *, PpkA phosphorylation of Fha can override TagF repression [59]. Recently, the phosphorylation of tyrosine 139 of TssC in *Francisella tularensis* has been shown to occur and weakens the stability of sheaths showing the existence of additional mechanisms of phosphorylation control [60]. Since these systems function at the protein level, they facilitate rapid transitions from unassembled to assembled T6SSs to immediately respond to activatory stimuli. Moreover, the activating role of PpkA and repressive roles of PppA and TagF may also be required for the ordered assembly of the T6SS nanomachine and to introduce a delay in T6SS re-assembly for spatial re-orientation towards target competitors. Thus, we anticipate that additional T6SS post-translational regulatory systems will be discovered in the years to come.

### The tail: a rigid tube encased in a contractile sheath

After the baseplate is assembled and coupled to the MC, it triggers polymerization of the phage tail-like structure that consists of the inner tube composed of stacked Hcp hexameric rings and the surrounded contractile sheath (Fig. 1iii, iv). The assembly of these structures is coordinated by TssA and they do not form in its absence [23, 61]. TssA associates with the BP and helps to recruit and incorporate new Hcp rings and sheath proteins at the end of the growing tail [23, 62]. The T6SS tail polymerization process is similar to that of major tail proteins in bacteriophages. For instance, in the T4 phage, the gp27 protein is the hub for the assembly of the bacteriophage tail tube composed of gp19 hexameric rings [19]. Likewise, the gp27-like ring domain of the VgrG trimer is the assembly hub for binding and polymerization of the T6SS inner tube composed of Hcp hexameric rings [19, 61]. Unlike phages, the T6SS gp27-like protein is fused as a single polypeptide to the gp5-like spike, forming the chimeric protein VgrG that allows penetration of the target cell [19, 63–65]. Some VgrG proteins, sometimes referred to as evolved VgrGs but now more commonly termed specialized VgrGs, contain additional C-terminal domains that are commonly antimicrobial and/or antieukaryotic effectors or adaptors that secure the binding of specific effectors to the VgrG spike (Figs 1 and 2). Thus, VgrG proteins are also docking sites for effectors prior to translocation [63, 66–69]. The VgrG spike is sharpened by a PAAR repeat superfamily protein that forms a conical structure (Figs 1–3). This shape forges the spike of the T6SS spike complex and can also be involved in loading effector domains to the puncturing structure (Fig. 2) [70]. In *V. cholerae* and *Acinetobacter baylyi *, the PAAR domain is essential for T6SS-dependent secretion [70]. In contrast, in *A. tumefaciens,* the absence of the PAAR protein diminishes but does not completely abolish the secretion of either the Hcp protein or the Tde and Tae effectors [69]. The completed baseplate consisting of the TssKEFG-VgrG/PAAR complex initiates polymerization of the inner tube from the base of the VgrG hub and associates with TssA, which recruits and incorporates new Hcp and TssBC rings to the end of the elongating tail (Fig. 1). The polymerized T6SS inner tube is formed by stacked hexameric Hcp rings with sixfold symmetry and arranged in a head-to-tail configuration to conform an external diameter of ⁓80 Å and an internal lumen of 40 Å [12, 71]. To assemble the sheath that wraps the inner tube, TssBC blocks are incorporated at the terminal end of the developing tail. TssB and TssC assemble into helical tubules resulting from the stacking of 12-fold symmetry cogwheel-like structures with an internal diameter of 80–100 Å, which is sufficient to fit an Hcp tube (⁓80 Å) [72]. The atomic structure of the sheath subunits has been resolved in *V. cholerae *, *F. tularensis* and *P. aeruginosa* [24, 73, 74]. The native contracted sheath forms a right-handed six-star helix stabilized by a core domain assembled by four β strands. The T6SS sheath subunits are evolutionarily related to the gp18 T4 phage sheath. Specifically, the inner and middle layer structures of the T6SS sheath are conserved with those of other contractile phage sheaths; however, the structure of the outer layer is distinct [24].

Upon activation by an unknown signal, the sheath swiftly contracts into a state of decreased energy, propelling the Hcp tube, the VgrG-PAAR spike, and the corresponding effectors into neighbouring target cells or the extracellular space (Fig. 1iv-v). Comparisons with the phage suggest that the BP is responsible for triggering the contraction of the sheath upon propagation of a signal likely generated in the MC [75]. The contraction proceeds rapidly and concentrically in a coiled-spring-like mechanism from the BP, with the TssB/TssC handshake interactions acting like hinges, facilitating the transition and enabling the sequential movement of adjacent subunits within the sheath. During contraction, there is a 45° clockwise rotation about the perpendicular axis of the helix. This results in ⁓30 % shortening and ⁓50 % widening of the extended sheath structure and a slight increase in the channel width (Fig. 1v) [25, 74, 76]. Molecular modelling against structurally related R-type pyocins, T4 phage and a cryo-EM structure of a non-contractile sheath mutant, supports a model whereby a conserved α-helix, present in TssC in the T6SS sheath, interacts with the Hcp in the extended conformation [24, 25, 72–74, 76, 77]. Upon firing, the sheath lattice rapidly pulls towards the membrane, twists and expands, pulling away from the Hcp interaction and at this point likely transferring some force. More importantly, the pre-contracted TssC subunits further along the sheath are thought to remain in contact with Hcp helping to facilitate this transfer of force and rotation into the Hcp inner tube driving it through the membrane complex and out of the cell. A TssA cap protein at the end of the sheath may further improve force transfer [23, 62]. The contraction of the sheath is coupled with the translocation of the inner tube and the spike to deliver effectors (Fig. 1iv-v).

### ClpV and T6SS sheath recycling

The contraction of the sheath results in conformational changes that expose a specific domain of TssC that is recognized by ClpV (Fig. 1vi–vii). ClpV is an ATPase family protein that provides the energy and biochemical interactions required to disassemble contracted sheaths after T6SS firing to recycle TssB and TssC components [25, 29]. Historically, this ATPase was proposed to be the energy source that facilitates the translocation of T6SS-dependent substrates [12]; however, [78] demonstrated that ClpV actually dismantles a tubular structure made of VipA (TssB) and VipB (TssC) subunits. This structure was later identified as the contractile sheath that surrounds the inner tube [25, 29].

ClpV belongs to the Hsp100/Clp family of proteins within the ring-forming AAA^+^ superfamily of ATPases (ATPases associated with diverse cellular activities). Interestingly, the V in ClpV stands for ‘virulent bacteria’, since this protein family was first identified in bacteria interacting with eukaryotic cells, including human pathogens such as *Salmonella* Typhimurium and Enterohemorrhagic *E. coli* (EHEC) [79]. Currently, it is well established that *clpV* genes are found specifically in T6SS clusters and are distributed among pathogenic and non-pathogenic bacteria [10, 14]. Within the Hsp100/Clp family, ClpV subfamily proteins are most similar to the ClpB protein subfamily; however, they cluster in a separate group with a marked phylogenetic distance [79]. Unlike other Clp proteins, ClpV is not associated with a proteolytic subunit and is therefore not involved in proteolysis [79]. This is consistent with its role in the disassembly of the contracted sheath.

ClpV has an N-terminal domain and two AAA domains, defined by a conserved sequence of 200 to 250 amino acids, including the Walker A and Walker B motifs responsible for the binding and hydrolysis of ATP, respectively. ATP hydrolysis induces conformational changes in AAA^+^ proteins, providing the force required to unfold or disassemble substrates [80]. According to Bönemann *et al*. (2009), a particular variation of the ClpV protein containing mutations in both Walker B motifs exhibits inadequate ATP hydrolysis, resulting in a malfunctioning T6SS. This observation suggests that the disassembly of the TssBC sheath complex is intricately linked to the ATP hydrolysis activity within ClpV. Indeed, the AAA domains of ClpV are responsible for protein oligomerization into hexameric assemblies that form a two-layer structure with an internal pore that is essential for substrate recognition and processing. The N-terminal domain of ClpV is connected to the AAA^+^ ring via a flexible linker. The N-domain is structurally mobile, which facilitates binding to the contracted sheath to mediate substrate specificity and to lead the complex to the pore for processing. ClpV targets the N-terminal helix of TssC, which is exposed in the outer layer of the contracted sheath [80, 81]. A single ClpV hexamer occupies 1.5 dodecameric repeat units of TssBC, representing a binding ratio of 1 : 3 (ClpV:TssC) [80].

The aforementioned divergence in the structure of the sheath outer layer can be explained by the fact that this region is where the interaction between the ClpV unfoldase and the T6SS sheath occurs. Because phages or R-type pyocins do not recycle their sheaths, they do not interact with ClpV in this region. In *P. aeruginosa *, the interaction of ClpV1 with the sheath is enhanced by the accessory component TagJ, which has been suggested to recruit the ATPase to the sheath structure through interaction with TssB1 [82]. As described above, TagJ has been shown to stabilize the sheath from the baseplate during assembly [30]. Thus, the interaction of the accessory component TagJ with ClpV suggests that it also assists to locate the recycling system for a suitable disassembly process. Interestingly, some differences in the oligomerization behaviour of ClpV proteins were reported in early studies. In these, ClpV^ST^ (*S*. Typhimurium) formed oligomers in the absence of ATP, whereas the oligomerization of ClpV^EC^ (* E. coli *) was strictly dependent on the presence of nucleotides and oligomers were not observed in their absence. These initial observations were hypothesized to indicate striking diversity between the two ClpV proteins tested that reflected the sequence diversity of ClpV proteins [79]. Nowadays, it is known that ClpV proteins are key components of T6SSs and these systems clade into different phylogenetic groups. The different T6SS phylogenetic groups can have different ClpV forms. Specifically, the bacteria studied by Schlieker *et al*. [79], EHEC and *Salmonella* have T6SS clusters that belong to different T6SS phylogenetic groups (1 and 3, respectively) and thus have different ClpV proteins. Additionally, these distinct phylogenetic groups present structural differences in components such as TssA and the accessory components that stabilize the sheath (TagA/TagBJ), resulting in a different mode of assembly and, more importantly, in different T6SS dynamics [30]. The T6SS clusters from groups 1 and 2 present a long TssA that interacts with TagA to stabilize the sheath from the opposite side of the baseplate and represent systems with a long residence time before firing (slow systems). The T6SS clusters from groups 3, 4 and 5, such as *Salmonella *, *P. aeruginosa* and *P. putida *, contain a short TssA that stabilizes the sheath from the baseplate due to the interaction with the accessory proteins TagB or TagJ. These systems have a short residence time and are triggered immediately after the sheath reaches the other side of the cell (fast systems) [23, 30, 42]. Differences in the oligomerization of ClpV proteins may reflect differences in the performance of the recycling systems. In this line, the absence of ClpV has different effects among T6SS^+^ bacteria. For instance, ClpV is not essential in *V. cholerae *, where, although infrequent, single and productive rounds of T6SS firing occur in Δ*clpV* cells, which retain a degree of bactericidal activity [25, 29]. In contrast, the absence of ClpV1 from the H1-T6SS in *P. aeruginosa* completely abolishes the secretion of Hcp1 [12, 83]. Although in *Francisella,* the T6SS was initially shown to lack a *clpV* gene, subsequent studies have shown that this system requires ClpB for T6SS dynamic activity and phagosomal escape [84]. Thus, in cases where ClpV is present, it facilitates T6SS sheath disassembly and enables new rounds of assembly and T6SS firing from the same MC, thereby maximizing its activity (Fig. 1).

In summary, the T6SS puncturing structure is an exceptionally efficient protein nanomachine that facilitates the delivery of a wide variety of effectors. The following section of this review will dive into a detailed description of several of these, as after all, the effectors are the true mediators behind the functional outcomes of the T6SS.

### Diversity of T6SS effectors

T6SS effectors are proteinaceous substrates that mediate a variety of functions that benefit the expressing bacterium. The principal functions of T6SS effectors include compromised growth or outright killing of competitors, the acquisition of metal ions, and the manipulation of host cells. The manipulation of the host mainly includes destruction or subversion of eukaryotic cells by promoting invasion or suppressing the immune system. Many of these bacterial effectors have a specific function and tend to be enzymatic. In fact, the first identified T6SS effector, VgrG-1 from *V. cholerae *, cross-links actin and is considered a classical anti-eukaryotic 'virulence factor' that causes the collapse of the cytoskeleton in macrophages [1, 63, 85]. However, it is now clear that the primary role of the T6SS is interbacterial competition since antibacterial toxins are the most frequently observed T6SS effectors. The discovery and characterization of antifungal effectors and effectors that allow bacteria to scavenge important metal ions have expanded the role of T6SS effectors from being mainly antibacterial agents to having a wider range of target organisms (Fig. 3). Importantly, effectors that act as siderophores not only assist the producing organism but can also indirectly deprive competitors of metal ions in shared environments, thereby exerting a broader impact on microbial growth and competition. Some antimicrobial effectors can still promote virulence but in a less direct manner, for example, killing commensal organisms for access and hostile takeover of a particular host niche [86, 87]. Additionally, recent studies have shown effectors that promote biofilm formation, along with proteins encoded within T6SS clusters that mediate adhesion. These proteins may not necessarily be secreted by the T6SS but can still alter the behaviour of T6SS^+^ bacteria [88, 89]. These functions benefit T6SS-harbouring organisms by enhancing the effectiveness of the contact-dependent T6 system or by facilitating communication between bacteria that could result in beneficial group behaviours such as protection from predation.

Importantly, the T6SS can also be used as a delivery platform to target a variety of locations. This includes extracellular locations in a general one-step secretion mechanism directly in prey bacteria, as well as other micro-organisms such as fungi, and the cells of higher eukaryotes. Such attributes give the T6SS the capacity to mediate or target a wide range of processes or organisms that are beyond the capabilities of other secretion systems. This review cannot cover the complete spectrum of effector research since this is a very active field, but here we outline the general mechanisms of T6SS effectors and showcase some exciting recent examples.

### T6SS effectors according to their target cells

The T6SS effectors can be divided into two primary categories based on their targets, namely eukaryotic and prokaryotic cells. To date, T6SS eukaryotic targets include fungi and animals whilst the prokaryotic category includes Gram-positive but predominantly Gram-negative bacteria. Interestingly, effectors with activity against both prokaryotes and eukaryotes have been described and recognized as ‘transkingdom’ effectors [90]. Here, we draw attention to several recent examples from these categories.

#### Anti-eukaryotic effectors

##### (i) Fungal cell-targeting effectors

A breakthrough in our understanding of the capabilities of T6SS came in 2018 with a clear demonstration of T6SS-dependent killing of fungi. The T6SS of *S. marcescens* injects at least two effectors with activity against *Saccharomyces cerevisiae, Candida glabrata* and *Candida albicans* [2]. The first described T6SS antifungal effector, Tfe1, disrupts the plasma membrane potential, leading to fungal death. The second identified antifungal toxin, Tfe2, triggers a large nutrient imbalance with disrupted nutrient uptake, altered amino acid profiles and provokes activation of autophagy in fungal cells [2]. Fungal killing has also been demonstrated in *Klebsiella pneumoniae,* with a specialized effector protein capable of intoxicating bacteria and yeast [91]. This effector with activity against prokaryotes and eukaryotes is considered the first ‘transkingdom’ effector (Fig. 3) [90]. It is highly likely that many more examples of effectors with activity against fungal pathogens will be identified in the near future. Indeed, this has been speculated in a brief review by [92] that we encourage the reader to refer to for further insights.

##### (ii) Animal cell-targeting effectors

Most T6SS anti-eukaryotic effectors target animal cells. Due to space constraints, we refer interested readers to recent reviews that focus on cell-animal targeted-eukaryotic effectors [7, 8]. Three compelling examples of antieukaryotic effectors are discussed below.

Research on *F. tularensis* and its evolutionarily divergent T6SS^ii^ group is generating significant findings, with different structural components and multiple effectors identified in this Gram-negative pathogen [84, 93]. As an example, the effector PdpC plays an important role during infection by allowing bacteria to escape the phagolysosome and thus enabling intracellular replication [84, 94, 95]. PdpC is also required for the attachment and subsequent invasion of *F. tularensis* into red blood cells [erythrocytes] [96]. Using PdpC-gfp fusions, the authors showed that this effector is secreted into erythrocytes during invasion and accumulates in a defined area [96]. Interestingly, *pdpC* has expression heterogeneity as only a subset of *F. tularensis* cells expressed PdpC within the population. Nevertheless, the translocation of a T6SS effector tagged with gfp is an interesting development, as visualizing the delivery of T6SS effectors is extremely difficult. This innovation provides promising prospects for future research using high-resolution microscopy.

A second notable effector linked to the invasion of eukaryotic host cells is *P. aeruginosa* TseL*,* TseL^PA^ [97]. This effector possesses a lipase domain from class 3 (cd00519) and belongs to the Tle2 family of T6SS lipases [98]. Encoded within the genetic island of VgrG7, TseL^PA^ is T6SS delivered and predicted to be secreted by the H2-T6SS although not experimentally demonstrated [97, 98]. Similar to other T6SS lipases, TseL^PA^ breaks down lipids, has anti-microbial and anti-eukaryotic activity and mutation of the catalytic site, H466A histidine, abolished both activities [97]. Encoded upstream of *tseL* are two putative immunity genes: *tsiP1* and *tsiP2*. The expression of the immunity proteins, TsiP1 or TsiP2, provides significant protection to periplasmically targeted TseL^PA^, with TsiP1 conferring the highest level of protection [97]. The deletion of *tseL* significantly reduced the competitive advantage of *P. aeruginosa* strain YSZa7 in intraspecies competition against PA14; and the competitive advantage of PAO1 in interspecies competition against *E. coli* and *A. baumannii *. This deletion also decreases its invasion capacity into eukaryotic epithelial cells but results in a ~tenfold increase in phagocytosis into macrophage cells, demonstrating that TseL^PA^ plays an antiphagocytic role [97]. Since lipase toxins are prevalent in T6SS systems and a single bacterium may possess multiple homologues, additional research is necessary to elucidate their distinct impacts; especially as they are often transkingdom effectors.

The third antieukaryotic effector we highlight here has been recently characterized by the Bengoechea research group. This effector is a specialized VgrG, named VgrG4, from *K. pneumoniae *. As previously noted in this review, VgrG4 exhibits antimicrobial activity against both bacterial and fungal species [91]. The microbial toxicity is due to the DUF2345 domain of VgrG4 that triggers the induction of reactive oxygen species (ROS) [91]. In addition to the antimicrobial activity, VgrG4 subvert human cells by targeting mitochondria [99]. This is fascinating, as mitochondria are themselves evolutionally descended from bacteria, but are now specifically dependent upon eukaryotic cells. In this case, VgrG4 results in the transfer of Ca^2+^ from the endoplasmic reticulum to the mitochondria, activating a protein that triggers mitochondrial network fragmentation, Drp1 [99]. Elevated Ca^2+^ levels also activate the innate immune receptor NLRX1, which limits the activation of inflammatory responses in a ROS-dependent manner. This process is exploited by the pathogen to evade innate responses and promote infection [99]. Of note, the DUF2345 or ROS toxic domain (RTD) is also present in several VgrG proteins of *E. coli *, *P. aeruginosa* and *A. baumannii,* which suggests that the RTD-containing VgrG protein might share similar functions in other bacteria [99]. In summary, some *K. pneumoniae* strains contain a T6SS and VgrG4 that they deploy to combat microbial competitors and enable this pathogen to evade innate immune responses.

Thus, the latest research has revealed new functions for the T6SS and its effectors in mediating fascinating biological functions, manipulating host cells and acting as bacterial virulence factors. Moreover, the increasing frequency of transkingdom effectors suggests that we are only scratching the surface of what these systems can do. An important missing element in understanding the biology of T6SS and eukaryotic effectors is that currently there are no identified T6SS effectors that specifically target plant cells. However, such effectors are anticipated to exist given the apparent role of T6SSs in facilitating interactions between rhizobacteria and plants [100]. In fact, one of the first reports of T6SS described the importance of this secretion system for the successful nodulation of leguminous by rhizobia species [101] (Fig. 3).

### Antibacterial effectors

Antibacterial effectors play a crucial role in the competitive interactions between bacteria, as they are responsible for killing or inhibiting the growth of rival bacteria. They are the most frequent and studied type of effectors associated with the T6SS. A critical concept here is that antibacterial effectors are encoded as toxin immunity pairs in the genome. However, as many of the immunity-encoding genes are small, they are frequently missed in genome annotations. The presence of these immunity genes, commonly downstream of those encoding putative toxic effectors, enables researchers to target initial experiments towards functional characterization in bacteria as opposed to eukaryotic cells. The mechanisms by which antibacterial effectors exert their activity vary, but they generally involve targeting essential cellular processes in the recipient bacteria. Classically, single effector proteins have been associated with single targets or specific functions. The T6SS-targeted molecules are involved in the synthesis or maintenance of critical cellular structures, including the cell wall, the membrane, nucleic acid molecules and proteins; thus, they are crucial for bacterial fidelity or survival. The diverse range of effectors reflects the multitude of target molecules they interact with: amidases that target the peptidoglycan cell wall, membrane-degrading lipases, effectors that disrupt protein biosynthesis, toxins that degrade energy metabolism intermediates (e.g. ATP/NADP^+^), proteases, pore-forming toxins, which dissipate proton motive force, effector that increase cellular permeability, toxins targeting bacterial cell division, nucleases targeting DNA/tRNA/rRNA, or deaminases that mutate DNA [102–114].

Several novel exciting examples of T6SS antibacterial effector biology have been revealed in recent years. In the following section, we initially focus on antibacterial effectors that target Gram-positive bacteria; then, we explore effectors that provoke genetic changes, effectors whose activity is influenced by the environment, a multifaceted effector that confers advantages to bacterial cells in different ways, and finally, we describe effectors that manipulate bacterial behaviour.

#### (i) Targeting Gram-positive bacteria

One of the long-standing dogmas of the T6SS was the inability of this system to target or kill Gram-positive organisms. This dogma was challenged in 2019 by Molina-Santiago *et al.,* whom described that the *Pseudomonas chlororaphis* T6SS leads to higher rates of sporulation of the Gram-positive bacteria *Bacillus subtilis* [115]. Recently, Le and co-authors [116] have shown that *A. baumannii* secretes d-lysine resulting in an increased extracellular pH and enhancing the peptidoglycanase activity of the T6SS effector Tse4. Excitingly, this dual combination is able to outcompete both, Gram-negative and Gram-positive bacteria including *Staphylococcus aureus *, *B. subtilis* and *Listeria monocytogenes* with Tse4 harbouring both lytic transglycosylase and endopeptidase activities [116]. Importantly, the intoxication of the Gram-positive bacteria is T6SS^+^ contact-dependent since the topical addition of Tse4 does not result in *B. subtilis* death [116]. *B. subtilis* is also killed by the plant pathogen *Acidovorax citrulli* in a T6SS contact-dependent manner together with a panel of other organisms including *E. coli *, *Enterobacter cloacae *, *P. aeruginosa *, *Mycobacterium smegmatis *, as well as fungal competitors such as *C. albicans*, *S. cerevisiae* and *Pichia pastoris* [117]. Among the multiple putative T6SS effectors identified in this strain, RhsB and RhsE had the strongest impact against *E. coli* and *B. subtilis* [117]. *B. subtilis* cells expressing the RhsB cognate immunity proteins RimB1 or RimB2 are protected from growth suppression [117]. This indicates that the effector RhsB can enter Gram-positive cells to mediate its function and is blocked by the cognate immunity proteins. These examples represent significant developments in the field of T6SS-effector targeting Gram-positive organisms, with certainly more breakthroughs on the horizon.

#### (ii) Effectors mediating killing and driving evolution

The capacity of a single T6SS antimicrobial effector to induce genetic changes in recipient cells, rather than only nucleotide cleavage leading to death, has recently been demonstrated. The effector is a double-stranded DNA deaminase A (DddA) from *Burkholderia cenocepacia.* DddA has been shown instrumental to kill several bacterial species including *P. aeruginosa *, *P. putida* and other *Burkholderia* species through chromosomal degradation and DNA replication arrest [110]. In bacteria resistant to DddA killing, this effector assumes the role of a powerful and direct mutagen. Its mechanism of action involves the deamination of cytosine in double-stranded DNA, resulting in its conversion to uracil [110, 112]. These C-to-U transitions occur throughout the genome and accelerate the acquisition of rifampicin-resistant mutants through changes in the *rpoB* gene that encodes the β-subunit of the RNA polymerase. This finding demonstrates that T6SS bacterial effectors can drive adaptions, expanding the impact of T6SS on the target population beyond T6SS-dependent growth suppression or death.

#### (iii) Conditional toxicity

The activity of some T6SS effectors, such as *P. aeruginosa* Tse4 (Tse4^PA^), is influenced by the environment in which interbacterial competition occurs. This pore-forming effector disrupts the ΔΨ component of the proton motive force and, importantly, displays greater activity in high-salt environments [118]. Tse4^PA^ works synergistically with effectors that compromise the cell wall (Tse1 and Tse3) or inactivate intracellular electron carriers (Tse6) amplifying the impact of T6SS-independent effectors [118]. Two important elements are considered here: first, the efficacy of the effectors is not determined solely by their expression but is also influenced by their activity; second, two (or more) effectors can work synergistically and have an amplified impact beyond the effect of the individual effectors working independently.

#### (iv) The all-in-one effector

A multifaceted effector that has been recently characterized is TeoL (T6SS effector for recruitment of OMVs via LPS) that is secreted by the T6SS1 of *Cupriavidus necator,* a member of the Burkholderiales family. *C. necator* T6SS1 is repressed by Fur and derepressed under low iron conditions, leading to extracellular secretion of TeoL [119]. Extracellular TeoL binds to the surface of outer membrane vesicles (OMVs) by interacting with the O-antigen of LPS present on OMVs from both related and unrelated species [119]. Subsequently, TeoL facilitates binding to recipient cells through interaction with the OM receptors CubA and CstR tethering the OMV to the cell surface [119]. This results in the delivery of OMV composed of phospholipids, lipopolysaccharides (LPSs) and OM proteins and can encapsulate a luminal cargo of periplasmic and cytoplasmic components such as proteins, peptidoglycan, quorum-sensing (QS) molecules, nucleic acids and metal ions to recipient cells through an unknown mechanism. Remarkably, this delivery confers advantages to T6SS^+^ bacterial cells in four ways [119]. The first, it acts as a siderophore to promote iron acquisition to enhance growth in iron-limited conditions. The second, it enhances competitive growth against *Yersinia pseudotuberculosis* that required both TeoL and a functional T6SS. The third, it increases uptake of OMVs facilitated by the TeoL effector, which confers *C. necator* with increased resistance to oxidative stress (H_2_O_2_). Interestingly, TeoL OMV uptake requires the surface receptors CubA or CstA to increase resistance to oxidative stress. This suggests that TeoL is an active component of the OMV that contributes to increased resistance and emphasizes the requirement of its delivery to the cell, rather than simply being present in the environment. Finally, the presence of TeoL enhanced plasmid transfer mediated by OMVs. Higher numbers of transformants were obtained after incubation with OMVs isolated from *C. necator* in contrast to those derived from the Δ*teoL* strain [119]. Thus, this effector operates essentially as a siderophore that facilitates iron acquisition. However, due to the mechanism of binding to OMVs and facilitating their entry, the benefits of this effector are amplified through competition, protection against oxidative stress and horizontal gene transfer. Additionally, the fact that TeoL can bind to LPS of different species improves the chances that *C. necator* can obtain all the above-mentioned benefits from its investment in the T6SS and TeoL secretion.

#### (v) Manipulating bacterial behaviour

*P. aeruginosa* TepB effector, T6SS effector promoting biofilm formation, is involved in the manipulation of bacterial behaviour [89]. This effector is delivered by *P. aeruginosa* H3-T6SS and is responsible for three different phenotypes, namely biofilm formation, motility and stress resistance. Indeed, deletion of *tepB* results in reduced biofilm growth, diminished swimming and swarming motility and attenuated resistance to oxidative, osmotic and acid stresses [89]. This suggests that TepB acts as an inter-microbial signal effector, a role for a T6SS effector that has only been suggested for the T6SS but elegantly demonstrated for other competition systems such as the T5SS CDI systems [120].

#### (vi) Contact independent effectors

A particularly interesting T6SS effector protein is Tce1 that is a Ca^2+^ and Mg^2+^-dependent DNase [121]. Tce1 can be delivered in a standard T6SS contact-dependent mechanism by the T6SS-3 of *Y. pseudotuberculosis.* As expected, killing is suppressed in prey cells expressing the corresponding immunity protein, Tci1. Intriguingly, Tce1 also mediates a competitive advantage in liquid medium, that has reduced cell contact, and across bacterial cell impermeable barriers that is dependent on a functional T6SS-3. Thus, Tce1 is a non-canonical T6SS effector that can mediate a contact-independent mechanism of bacterial killing [121]. Entry of Tce1 requires binding to the outer membrane proteins OmpF and BtuB, as well as TonB, for transfer across the membrane of prey cells in a mechanism similar to some bacteriocins.

A second contact-independent *Y. pseudotuberculosis* effector has been recently characterized and named CccR [122]. This effector has an N-terminal FIC domain involved in post-transcriptional modification by AMP addition of target proteins and a DNA-binding C-terminal domain that acts as a transcriptional factor. When inside prey *E. coli* cells, the FIC domain AMPylates the cell division protein FtsZ, resulting in cell filamentation and growth arrest. Contrastingly, when expressed in the donor, *Y. pseudotuberculosis,* the C-terminal helix-turn-helix domain autoregulates and represses the *cccR* promoter, akin to that observed for some toxin-antitoxin systems [123]. When CccR is translocated into recipient *Y. pseudotuberculosis* cells it also acts as a transcription factor repressing the *cccR* gene. Remarkably, in these kin recipient cells, CccR has a broader impact, with 447 genes up- or down-regulated more than 1.2-fold with an enrichment for genes whose products control bacterial behaviours including nutrition acquisition, motility and energy production. This demonstrates that CccR acts as an intercellular transcriptional regulator that mediates cell-to-cell communication and coordinates bacterial behaviour after it has entered target recipient cells. Strikingly, this T6SS-dependent effector can also be delivered in a contact-independent manner with uptake, requiring the target cells to have the surface-expressed TonB-dependent siderophore receptor ferric hydroxamate uptake A (FhuA) that usually serves as the outer membrane receptor for uptake of ferrichrome [122]. It is interesting to consider that the presence of longer-range effects, specifically contact-independent killing, in two of the T6SS effectors in *Y. pseudotuberculosis* is likely the result of evolutionary selection. However, these two articles present a significant challenge to the widely accepted dogma that T6SS effectors operate exclusively through contact-dependent killing. Instead, these studies suggest that previously identified T6SS effectors should be assessed for roles in mediating non-contact-dependent killing, growth suppression and even intracellular signalling.

### Delivery of T6SS effectors

#### Cellular compartments targeted by T6SS effectors

T6SS toxins can act in different cellular compartments, such as the cytoplasm or the periplasmic space of bacteria. In multiple instances, the location of an effector is indicated by the location of its cognate immunity protein. For example, immunity proteins can carry signal peptides that direct their transit across the inner membrane to the periplasm, indicating that their cognate effectors function in this cellular compartment. Classical examples of such effectors include amidases that target peptidoglycan and lipases that target membrane lipids [113, 124].

A significant development in understanding the functions of the T6SS was the demonstration that this secretion system is not only capable of delivering effectors directly into prey cells but also acts as a nanomachine for general secretion in the extracellular environment. This was effectively demonstrated with a range of effectors that T6SS^+^ bacteria can use to acquire metal ions, including YezP from *Y. pseudotuberculosis* that has affinity for zinc, TseM from *Burkholderia thailandensis* that binds extracellular manganese ions, TseF present in *P. aeruginosa* responsible for facilitating iron acquisition, and Azurin that can be used for copper scavenging amongst others [4, 109, 125, 126]. In ion-limited environments, these effectors interact with surface proteins to facilitate the uptake of ions to confer a competitive advantage or promote survival in challenging environments such as host niches that are often ion-restricted or hostile.

An intriguing development in metal effector biology has been the demonstration that ion-sequestering effectors can also act intracellularly in eukaryotic cells. Additionally, these effectors can be extremely short proteins, e.g. TssS from *Y. pseudotuberculosis* has 48 amino acids. This micropeptide is mostly delivered via the T6SS4 into host cells to enhance its virulence and dampen the immune response [127]. Via direct Mn^2+^ sequestration, this effector counteracts the cytoplasmic Mn^2+^ increase and inhibits the immune response mediated by the stimulator of interferon genes (STING) against *Y. pseudotuberculosis* [127]. Supporting this, TssS-mediated STING inhibition prevents bacterial clearance in mice. It is exciting to speculate on the potential applications of other ion chelating effectors in other contexts, including both, host–pathogen interactions and their delivery into competing microorganisms. This is especially important considering the limitation imposed by metal ions, particularly iron, on many bacteria. Moreover, the fact that TssS is an extremely short effector protein suggests that similar effectors might have been overlooked in other bacterial species since short proteins tend to go unnoticed during annotation.

#### Loading effectors

To ensure the effective loading of effectors to the structural components of the T6SS machinery (such as Hcp, VgrG and PAAR), a variety of different proteins are required to guide, stabilize and protect them. Certain effectors are encased in a protective shell structure (Rhs) for loading to the system, whilst others require chaperones and/or adaptors. In this section, we highlight the most relevant mechanisms for T6SS effector loading.

##### (i) T6SS Rhs effectors

The T6SS field has witnessed a significant increase in Rhs effector research over the past few years. *rhs* (rearrangement hot spot or recombination hot spot) genes were first defined as recombination-prone, imperfectly repeated regions of the *E. coli* chromosome [128]. Homologous of these genes were broadly observed in other bacterial genomes and they feature a conserved central region and variable 5′ and 3′ regions [129]. The central region enabled homologous recombination and lateral displacement of the 3′ end leading to strain diversity through HGT, but the evolutionary driver for this diversity was not initially clear [129]. Insight came with the first characterization of an Rhs protein playing a role in interbacterial competition. That is, the 3′ region of an *rhs* gene (*rhsB-CT^3937^*) from *Dickeya dadantii* that encoded a C-terminal toxin tRNase domain and the downstream gene, an immunity protein [130]. In this example, the 5′ of the Rhs protein encoded a T5SS contact-dependent inhibition (CDI) passenger domain that enabled cell surface expression and transfer of the C-terminal toxic effector domain to inhibit the growth of *E. coli* prey cells [130]. Subsequent bioinformatic analysis suggested that *rhs* genes frequently encoded C-terminal putative polymorphic toxin domains, with the most frequent being predicted nucleases [130, 131]. Thus, the 3′ recombination and acquisition of DNA encoding a new toxin domain, along with the downstream gene encoding the cognate immunity protein, can displace the existing 3′ modules and be evolutionarily selected for. The less frequent replacement of 5′ regions allow these effectors to be transported by different secretion systems such as the T5SS CDI and the T6SS.

The T6SS-related *rhs* genes are frequently located downstream of *vgrG* or chaperone genes (*eag*, *tec*/*tap*) and often encode an N-terminal PAAR/PAAR-like domain or other VgrG-connecting domains like the VgrG-interacting Rhs N terminus (VIRN) [132, 133]. The first characterization of VgrG-dependent Rhs proteins playing a role in interbacterial competition was observed in *D. dadantii *, where two effectors were identified, RhsA and RhsB, both encoding C-terminal DNase domains [134]. Following work in *P. aeruginosa* showed that Rhs proteins can have PAAR or PAAR-like DUF4150 domains for coupling to specific VgrGs for H1-T6SS delivery and interbacterial killing [135, 136]. A study in *S. marcescens* broadened the scope of Rhs effectors by revealing their potential contribution to T6SS intraspecies competition. This study also confirmed that certain Rhs effectors require adaptors [137], which are described below.

Rhs proteins can undergo two autocleavages, one at the N-terminus and one at the C-terminus at specific sequence motifs on either side of the conserved core domain [132]. Rhs cleavage can expose domains of the effector necessary for interaction with VgrG proteins and the loading and secretion of the effector itself [132]. The conserved core domain of Rhs proteins comprises spiralling β-sheets and has been proposed to form a shell-like structure that likely protects the C-terminal toxin domain (Fig. 2) [138]. Beautiful structural work using cryo-EM has recently demonstrated that Rhs proteins indeed form β-barrel cage-like structure or ‘cocoon’ around the C-terminal toxin domain that is capped by plug regions [139, 140].

Structural studies of Rhs effectors have led to unanticipated findings, for instance, the proteolysis of the RhsP effector from *Vibrio parahaemolyticus* enables dimerization of two Rhs cages in an antiparallel dimer [141]. Mutations that specifically disrupted this dimerization resulted in significantly reduced interbacterial killing, suggesting that this conformation contributes to prey targeting or effector loading. The recent study by Günther *et al*. [139] also used a cleaved version of *P. protegens* RhsA to obtain EM images showing a dimeric structure of RhsA initially thought to be an artefact. These two examples suggest that dimerization of Rhs effectors post-proteolysis might be a widespread mechanism key for this class of effectors.

Thus, the latest research on Rhs effectors associated with the T6SSs is uncovering the diversity of C-terminal effector domains, their ability to target a broad range of organisms, the capacity of these genes to evolve by recombination after horizontal gene transfer, structural insights into different effector loading, and the distinct dynamics for effector delivery. Undoubtedly, there will be more exciting developments in this space that will advance the field.

##### (ii) T6SS chaperones and adaptors

Hcp, the structural constituent of the T6SS inner tube, was identified as the first T6SS protein to present chaperone activity. Its role in stabilizing small Hcp-loaded effectors also serves as a means to load them into the tube and facilitate their delivery either extracellularly or into target cells (Fig. 2) [142]. Tse2 is an excellent example, with mutational analysis showing that the inner face of the Hcp1 hexamer ring is required for Tse2 binding, stability and secretion [142]. Some specialized Hcp effectors have also been identified and characterized (Fig. 2) [143]. These are particularly prevalent within Enterobacteriaceae, with more than 350 Hcp-toxin fusions identified in 17 species from this family [143]. Five major classes of Hcp-toxins have been identified, namely, HNH-DNase, Colicin-DNase, DUF2235, Pyocin S3 and Papain-like peptidases [143]. We anticipate further studies in this under-explored area.

The next step forward in the biology of T6SS adaptor and chaperone proteins revealed a range of proteins that facilitate effector loading but do not impact the overall T6SS function as they are not structural proteins of the T6SS and are not secreted via the T6SS (Fig. 2). The key T6SS adaptors, Tec/Tap (DUF4123), EagR (DUF1795) and DUF2169 are described in the following section.

The first to be identified were the DUF4123-containing proteins, independently characterized in two separate laboratories and termed either type six effector chaperone (Tec) or type six adaptor protein (Tap) (Fig. 2) 2) [144, 145]. This family of proteins was shown to mediate the interaction between an effector and its cognate VgrG; for example, in *V. cholerae *, VgrG1 loading of TseL for secretion and interbacterial killing requires Tap-1/TecL [144, 145]. Subsequent work showed that these proteins can promote effector loading to the C-terminus of both VgrG and PAAR proteins [69, 146]. Tap proteins can also have additional binding partners or co-chaperones named co-Tec that help their function and promote their stability [146].

The second family of T6SS chaperones identified and characterized was the DUF1795-containing proteins. This family was named effector-associated gene (Eag) and was simultaneously characterized in *S. marcescens* and *P. aeruginosa* [107, 133, 137]. Eag chaperones facilitate the stabilization and promote the loading of certain specialized PAAR effectors onto VgrG trimers (Fig. 2).

In *A. tumefaceins* the type VI DNase effector 2, Tde2 contains a DUF4150 PAAR-like domain and has been demonstrated to require the protein Atu3641 for translocation and VgrG2-dependent delivery. Atu3641 harbours a DUF2169 domain [69]. Genetic analysis of Tde2 has uncovered related genes harbouring PAAR or PAAR-like DUF4150 domains. Importantly, all orthologues present an upstream gene encoding a DUF2169 domain highlighting its conservation and critical role in specific toxin delivery [69]. An important distinction is that these three classes of proteins (Tap/Tec, Eag and DUF2169) are thought to not be secreted and are proposed to be stripped off during effector loading or T6SS assembly (Fig. 1 and Fig. 2, iv,v blue area).

The genes encoding the aforementioned adaptor and chaperone domains serve as genetic markers for iterative searches to identify additional effectors. Bioinformatic analysis has also suggested the existence of additional putative adaptor/chaperone proteins, such as RK06147, that are disproportionately found in clusters encoding spike complexes or effector homologues [147].

It is important to note that when establishing rigid definitions for bacterial secretion systems, it is inevitable that some exceptions to the rules will arise, as elegantly discussed in a review authored by [148] regarding these systems. In the T6SS field, a recent example has challenged the established belief that T6SS chaperones or adaptor proteins are not secreted (with the notable exception of Hcp proteins, which are structural T6SS proteins that can chaperone certain effectors during delivery). Dar and co-authors showed a clear example of the secretion of a periplasmic-acting toxic effector VP1390 that was dependent on a second protein VP1388, which they termed a co-effector [149]. VP1388 and VP1390 are co-dependent on each other for interaction with the VgrG1 spike and T6SS-dependent secretion. This co-dependent nature required for T6SS secretion led to the definition of the term ‘binary effector module’. This work shows that some adaptor proteins can indeed be secreted via the T6SS and broadens the types of proteins that could be potential substrates of the system.

##### (iii) New mechanisms of effector loading and inhibition

Structural work focused on the VgrG spike of the T6SS of EAEC has revealed a mechanistic trick used to inactivate effectors in attacking strains. The cargo effector, Tle1^EAEC^ is a phospholipase dependent on the VgrG^EAEC^ for delivery. The VgrG has the classical gp27 and gp5 domains, followed by a C-terminal extension harbouring a DUF2345 and a transthyretin-like domain (TTR) that mediates the interaction with Tle1 [150]. A cryo-EM structure showed that the binding of the VgrG trimer with three Tle1 monomers results in a conformational change that inhibits their activity, likely through interaction with the gp5 domain [151]. The inactivation was confirmed by adding increasing amounts of purified VgrG to Tle1 that exhibited dose-dependent inactivation; as expected, addition of a truncated VgrG lacking the gp5, DUF2345 and TTR domains did not block the activity [151]. Thus, effector loading to its cognate VgrG renders this effector temporally inactive and suggests that the dissociation of VgrG-Tle1 is required post-delivery for effector activity (Fig. 2). This mechanism could mitigate self-intoxication in T6SS^+^ producer cells and it is plausible that similar mechanisms will be discovered for other effectors.

##### (iv) Does an effector-empty system fire?

Another developing concept in the literature has been the idea that effector loading is required for T6SS assembly and firing. Conceptually, this is appealing as it would maximize effectors loaded and prevent futile firing of the T6SS, saving energy and T6SS components. This ‘onboard checking mechanism’ has been proposed in *V. cholerae* [152], *A. tumefaciens* [153] and *V. parahaemolyticus* [154]. In *V. cholerae *, the physical presence of TseL, VasX and VgrG3, but not their activities, is crucial for T6SS assembly [152]. However, in *A. tumefaciens* the presence of effectors Tde1 or Tde2 is required for TssBC sheath polymerization [153]. Interestingly, in the case of *V. parahaemolyticus* the checkpoint is reliant on a single conserved effector rather than one of several loaded effectors as observed in the two previous examples [154]. Intriguingly, a single amino acid change in the DUF2345 domain of a VgrG protein from *A. baumannii* can impede T6SS assembly [155]. This change might disrupt proper folding or block the interaction between the trimer of VgrG proteins with a required PAAR protein or an effector molecule and suggests that the ‘onboard checking mechanism’ could be a widespread process. These studies demonstrate that VgrG proteins with loaded effectors are required for the assembly of the systems and reinforce the concept that the structure of the spike complex matters for baseplate and sheath formation.

### Benefits of the T6SS

The primary advantage of the T6SS is its ability to penetrate various targets through a ‘crossbow’ mechanism, without the need for specific receptors to recognize prey cells and internalize effectors. The second attribute of the T6SS is that the system can be customized to export a variety of cargo types as evidenced by the orphan islands encoding additional VgrG/PAAR/Hcp/effectors in the genomes of Gram-negative organisms. These genes were likely acquired by horizontal gene transfer at different times and their proteins may either be directly compatible for secretion, or their DNA sequence might recombine for coupling to existing genes encoding specific T6SS components to allow secretion. Some cargo and specialized effector domains exhibit homology with effectors of other polymorphic toxin delivery systems such as the T4SS, T5SS and T7SS [131, 156]. The third positive aspect to consider regarding T6SS is that during a single firing event, a combination of effectors that might have synergistic activities can be delivered at once. This amplifies the potency of each effector and/or broadens the range of organisms susceptible to T6SS attack. In addition, the transkingdom effectors that are functional against a wide range of prokaryotes and eukaryotes expand the targets that can be attacked in one firing event.

### Tethering to increase T6SS efficiency and engineering of the T6SS

Given that the T6SS is a firing mechanism, T6SS attachment to target cells would conceptually increase its ability to optimally deliver effectors. This attachment has been recently identified in *Vibrio fischeri *, where a lipoprotein selectively binds prey cells prior T6SS attack [157]. This large surface-located >380 kDa protein called ‘type VI secretion associated lipoprotein’, TseL, is encoded within the T6SS2. In viscous conditions, both TseL and the T6SS2 are upregulated. TseL enhances aggregation promoting efficient T6SS-mediated killing of prey cells [157]. TseL is also required for T6SS-dependent competition within juvenile squid, the natural host of *V. fischeri* where T6SS^+^ strains compete, indicating that the adhesion factor is active in the host. Thus, TseL forms a new parallel between the T6SS and phages, as the latter require phage tail fibres or receptor-binding proteins to selectively bind prey and engage with the surface of target cells for efficient firing, a role that TseL may facilitate [157].

An excellent study on that subject enhances tethering through engineering *E. cloacae* T6SS^+^ cells to express nanobodies on their surface that bind specifically to surface-expressed proteins present on prey cells [158]. The nanobodies guide attacker bacteria selectively toward prey cells with matching receptors in environments where contact mechanisms are less frequent, such as liquid medium or mice assays. In this way, engineered *E. cloacae* selectively targeted T6SS-susceptible micro-organisms such as *E. cloacae* lacking three T6SS immunity genes (*rhsIA*, *rhsIB* and *tai4*) or T6SS^-^ *E. coli* cells [158]. Efforts are taking place to engineer effectors or spike proteins for different functions [159] and it is likely that in the future we will be able to ‘arm’ commensal bacteria with specific effector complements. Clearly, customized T6SS is an active area of research that will likely produce exciting results and offer the potential for precise manipulation of host (plant, animal or human) microbiota. In the future, this could be particularly useful in treating patients or improving crop yield by manipulating their microbiota with a high level of accuracy and specificity.

### Identification of T6SS effectors

Effectors of the T6SS were first identified *in silico* as DNA extensions encoding addition domains at the end of *vgrG* genes (Fig. 3) [1]. Subsequently, antibacterial effectors were discovered through secretome analysis with Tse1, 2 and 3 from *P. aeruginosa* being the first to be identified [160] and shown to be encoded in tandem with immunity-encoding genes. The identification of PAAR proteins accelerated the discovery of additional T6SS effectors. The genes encoding this newfound structural component are genetically linked to sequences encoding additional domains with predicted toxin functions or located in the proximity of putative effector encoding genes [70]. Increasingly genomic analysis and the systematic search for effectors in the genomic vicinity of known T6SS structural components, particularly *vgrG*, *PAAR* and *hcp* genes, are increasingly providing new insights in this field [34, 161–164]. Such effector encoding genes are less conserved than the core T6SS genes and remarkable variations can be observed at these loci including a wide range of GC contents, reflective of horizontal acquisition [131, 165]. Genes encoding *rhs* proteins with PAAR/DUF4150/DUF4280 and sometimes cryptic PAAR-like domains are commonly observed in T6SS^+^ bacteria [70, 135, 136, 166]. In addition, new motifs that can be used as markers for subclasses of T6SS effector genes have been identified, such as the Marker for type sIX effectors (MIX), Found in type sIX effector (FIX), aRginine-rich type sIX (RIX) [167–169] as well as genes encoding for the above-mentioned chaperone proteins. Transcriptomic approaches using isogenic mutants and profiling for co-regulation can also suggest *vgrG* islands encoding effectors that are coordinated with specific T6SSs in organisms where multiple separate T6SS clusters are present [170]. Recent reports show that certain effectors are part of the core genome and can be found in all isolates of a particular bacterial species. In contrast, other effectors are much more variable in terms of their presence or absence across different isolates [98, 171, 172]. Effectors that are not present in the core genome are likely recent acquisitions that may have beneficial functions in particular environments or play a role in interstrain competition, which could lead to an increased frequency within a population over time. Arguably, from an evolutionary perspective, the closer two organisms are related, the more likely it is that they compete for resources. Thus, killing or inhibiting close-related organisms provides the greatest benefit and would favour the acquisition and deployment of these genes in competitive niches. With the T6SS present in 25 % of Gram-negative bacteria [173] surely more systems and their effectors remain to be identified and characterized in the future and will contribute to this active research field.

## Future perspectives and conclusions

During the past two decades, there has been a substantial increase in knowledge about the T6SS since its initial discovery and characterization 17 years ago [1, 12]. The productive research on T6SSs has greatly expanded our understanding of bacterial warfare and opened new avenues for the development of novel antimicrobial therapies that target pathogenic bacteria. These systems can also act as direct virulence factors to subvert eukaryotic cells and kill fungal competitors. In addition, T6SSs have also been found to play an important role in beneficial relationships between bacteria and eukaryotic organisms such as the biocontrol of plant pathogens [14, 34, 161, 174, 175], highlighting their potential for biotechnological applications in the field of agriculture [176].

Biochemical and structural investigations have propelled our understanding of the T6SS forward. Parallels and contrasts with phage biology have yielded great insights into T6SS structure and dynamic. Thanks to these investigations, we now have a clearer picture of the subcomplexes and the stepwise assembly process of the high-energy structure of extended T6SSs (Figs 1–3). Future research will likely reveal a greater understanding of baseplate assembly and the mechanism that holds the T6SS structure in an extended high-energy state whilst the tube and sheath form. Developments in effector loading into the Hcp ring or onto the spike complex are continuing to expand our understanding of the range of components that can be coupled for delivery. Important questions remain regarding space restrictions and effectors loaded within the Hcp tube or accommodated on the spike complex once inserted into the MC. How are the adaptor and chaperone proteins stripped off? What triggers the firing of the T6SS? Where does the signal for firing initiate from? How is the signal transferred to trigger sheath collapse? The regulation of T6SS in bacteria at multiple levels, including transcriptional, post-transcriptional and post-translational control is also an area of expanding research [59, 125, 170, 177–182]. As mentioned earlier, post-translational regulation of T6SSs enables a rapid transition from inactive proteins to assemble T6SSs; these regulatory components are likely widespread, warranting further investigations in the future. The assembly of the T6SS structure fundamentally requires that core proteins are expressed in sufficient quantities at certain times [183]; for this to happen, there are many levels of control needed to be exerted by bacteria to result in the activation and deployment of these systems that we do not fully understand and need further investigations. Although the T6SS nano weapons are highly conserved with respect to core components and mechanisms of action, structural studies have only been conducted on selected model organisms due to technical constraints, expertise, chosen laboratory organisms and genetic tractability etc. However, the assembly of the T6SS machinery is a highly complex and fascinating process and to advance this field further, it is necessary to broaden research efforts beyond these few models towards investigating other bacterial species that display diverse T6SS structures. Doing so will enable an improved understanding of the complexity underlying T6SS structure while expanding our overall comprehension of this discipline. Thus, it is an exciting time for this field of research, with numerous opportunities to push the boundaries of our current knowledge.

Effectors are the true mediators of the T6SS function. This review covers the key elements of effector classes, their loading for delivery and captures recent developments on their functions. Increasing research on their targets is providing exciting insights into the biology of bacterial and eukaryotic cells. The remarkable knowledge gained from studying the well-explored type three secretion system (T3SS) and its effectors has led to major advancements in the understanding of host–pathogen interactions as well as fundamental insights into eukaryotic cell biology [184, 185]. It is exciting to speculate on the awesome biological knowledge that will come from understanding the T6SS in as much detail. In the same vein, the study of bacterial effector proteins and their targets is yielding fascinating insights into the biology of the different T6SS-targeted organisms. As bacterial effectors target the ‘Achilles’ heel’ or vulnerable points of bacteria, fungi or other eukaryotes, these findings open up new possibilities for the development of innovative strategies that could help overcome the growing problem of antibiotic and drug resistance. Antifungal effectors are especially exciting as current treatment options in this space are severely limited. Other outstanding questions include how are effectors selected for loading? Is this a random process? Can effectors be selected for loading that are best suited to meet a specific threat?

In conclusion, we look forward to the continuation of exciting developments in the structure/function and effector repertoire of the T6SS. Indeed, many of the truths we assign to the T6SS field depend on our current viewpoint which may change in the future as new discoveries are made in this area. We hope that one day, this system and its effectors will be understood and harnessed to be *killing in the name of*: designer microbes, personalized medicine, greener agriculture, therapeutic delivery and other functions yet to be conceptualized for the benefit of humanity and our planet.

## References

[1] Pukatzki S, Ma AT, Sturtevant D, Krastins B, Sarracino D (2006). Identification of a conserved bacterial protein secretion system in *Vibrio cholerae* using the Dictyostelium host model system. Proc Natl Acad Sci.

[2] Trunk K, Peltier J, Liu Y-C, Dill BD, Walker L (2018). The type VI secretion system deploys antifungal effectors against microbial competitors. Nat Microbiol.

[3] Yang X, Liu H, Zhang Y, Shen X (2021). Roles of type VI secretion system in transport of metal ions. Front Microbiol.

[4] Wang T, Si M, Song Y, Zhu W, Gao F (2015). Type VI secretion system transports Zn²⁺ to combat multiple stresses and host immunity. PLoS Pathog.

[5] Hernandez RE, Gallegos-Monterrosa R, Coulthurst SJ (2020). Type VI secretion system effector proteins: effective weapons for bacterial competitiveness. Cell Microbiol.

[6] Jurėnas D, Journet L (2021). Activity, delivery, and diversity of Type VI secretion effectors. Mol Microbiol.

[7] Monjarás Feria J, Valvano MA (2020). An overview of anti-eukaryotic T6SS effectors. Front Cell Infect Microbiol.

[8] Hachani A, Wood TE, Filloux A (2016). Type VI secretion and anti-host effectors. Curr Opin Microbiol.

[9] Bingle LEH, Bailey CM, Pallen MJ (2008). Type VI secretion: a beginner’s guide. Curr Opin Microbiol.

[10] Boyer F, Fichant G, Berthod J, Vandenbrouck Y, Attree I (2009). Dissecting the bacterial type VI secretion system by a genome wide *in silico* analysis: what can be learned from available microbial genomic resources?. BMC Genomics.

[11] Cherrak Y, Flaugnatti N, Durand E, Journet L, Cascales E (2019). Structure and activity of the Type VI secretion system. Microbiol Spectr.

[12] Mougous JD, Cuff ME, Raunser S, Shen A, Zhou M (2006). A virulence locus of *Pseudomonas aeruginosa* encodes a protein secretion apparatus. Science.

[13] Barret M, Egan F, Fargier E, Morrissey JP, O’Gara F (2011). Genomic analysis of the type VI secretion systems in *Pseudomonas* spp.: novel clusters and putative effectors uncovered. Microbiology.

[14] Bernal P, Llamas MA, Filloux A (2018). Type VI secretion systems in plant-associated bacteria. Environ Microbiol.

[15] Russell AB, Wexler AG, Harding BN, Whitney JC, Bohn AJ (2014). A type VI secretion-related pathway in Bacteroidetes mediates interbacterial antagonism. Cell Host Microbe.

[16] Bröms JE, Sjöstedt A, Lavander M (2010). The role of the *Francisella tularensis* pathogenicity island in type VI secretion, intracellular survival, and modulation of host cell signaling. Front Microbio.

[17] Spidlova P, Stulik J (2017). *Francisella tularensis* type VI secretion system comes of age. Virulence.

[18] Böck D, Medeiros JM, Tsao H-F, Penz T, Weiss GL (2017). In situ architecture, function, and evolution of a contractile injection system. Science.

[19] Leiman PG, Basler M, Ramagopal UA, Bonanno JB, Sauder JM (2009). Type VI secretion apparatus and phage tail-associated protein complexes share a common evolutionary origin. Proc Natl Acad Sci.

[20] Silverman JM, Brunet YR, Cascales E, Mougous JD (2012). Structure and regulation of the type VI secretion system. Annu Rev Microbiol.

[21] Rapisarda C, Cherrak Y, Kooger R, Schmidt V, Pellarin R (2019). *In situ* and high-resolution cryo-EM structure of a bacterial type VI secretion system membrane complex. EMBO J.

[22] Cherrak Y, Rapisarda C, Pellarin R, Bouvier G, Bardiaux B (2018). Biogenesis and structure of a type VI secretion baseplate. Nat Microbiol.

[23] Zoued A, Durand E, Brunet YR, Spinelli S, Douzi B (2016). Priming and polymerization of a bacterial contractile tail structure. Nature.

[24] Kudryashev M, Wang RY-R, Brackmann M, Scherer S, Maier T (2015). Structure of the type VI secretion system contractile sheath. Cell.

[25] Basler M, Pilhofer M, Henderson GP, Jensen GJ, Mekalanos JJ (2012). Type VI secretion requires a dynamic contractile phage tail-like structure. Nature.

[26] Aschtgen M-S, Bernard CS, De Bentzmann S, Lloubès R, Cascales E (2008). SciN is an outer membrane lipoprotein required for type VI secretion in enteroaggregative *Escherichia coli*. J Bacteriol.

[27] Felisberto-Rodrigues C, Durand E, Aschtgen M-S, Blangy S, Ortiz-Lombardia M (2011). Towards a structural comprehension of bacterial type VI secretion systems: characterization of the TssJ-TssM complex of an *Escherichia coli* pathovar. PLoS Pathog.

[28] Durand E, Nguyen VS, Zoued A, Logger L, Péhau-Arnaudet G (2015). Biogenesis and structure of a type VI secretion membrane core complex. Nature.

[29] Kapitein N, Bönemann G, Pietrosiuk A, Seyffer F, Hausser I (2013). ClpV recycles VipA/VipB tubules and prevents non-productive tubule formation to ensure efficient type VI protein secretion. Mol Microbiol.

[30] Bernal P, Furniss RCD, Fecht S, Leung RCY, Spiga L (2021). A novel stabilization mechanism for the type VI secretion system sheath. Proc Natl Acad Sci.

[31] Aschtgen MS, Thomas MS, Cascales E (2010). Anchoring the type VI secretion system to the peptidoglycan: TssL, TagL, TagP what else?. Virulence.

[32] Aschtgen MS, Gavioli M, Dessen A, Lloubès R, Cascales E (2010). The SciZ protein anchors the enteroaggregative *Escherichia coli* Type VI secretion system to the cell wall. Mol Microbiol.

[33] Santin YG, Camy CE, Zoued A, Doan T, Aschtgen M-S (2019). Role and recruitment of the TagL peptidoglycan-binding protein during type VI secretion system biogenesis. J Bacteriol.

[34] Bernal P, Allsopp LP, Filloux A, Llamas MA (2017). The *Pseudomonas putida* T6SS is a plant warden against phytopathogens. ISME J.

[35] Reglinski M, Monlezun L, Coulthurst SJ (2023). The accessory protein TagV is required for full Type VI secretion system activity in *Serratia marcescens*. Mol Microbiol.

[36] Weber BS, Hennon SW, Wright MS, Scott NE, de Berardinis V (2016). Genetic dissection of the type VI secretion system in *Acinetobacter* and identification of a novel peptidoglycan hydrolase, TagX, required for its biogenesis. mBio.

[37] Santin YG, Cascales E (2017). Domestication of a housekeeping transglycosylase for assembly of a Type VI secretion system. EMBO Rep.

[38] Scheurwater EM, Burrows LL (2011). Maintaining network security: how macromolecular structures cross the peptidoglycan layer. FEMS Microbiol Lett.

[39] Planamente S, Salih O, Manoli E, Albesa-Jové D, Freemont PS (2016). TssA forms a gp6-like ring attached to the type VI secretion sheath. EMBO J.

[40] Dix SR, Owen HJ, Sun R, Ahmad A, Shastri S (2018). Structural insights into the function of type VI secretion system TssA subunits. Nat Commun.

[41] Schneider JP, Nazarov S, Adaixo R, Liuzzo M, Ringel PD (2019). Diverse roles of TssA-like proteins in the assembly of bacterial type VI secretion systems. EMBO J.

[42] Santin YG, Doan T, Lebrun R, Espinosa L, Journet L (2018). In vivo TssA proximity labelling during type VI secretion biogenesis reveals TagA as a protein that stops and holds the sheath. Nat Microbiol.

[43] Brunet YR, Zoued A, Boyer F, Douzi B, Cascales E (2015). The type VI secretion TssEFGK-VgrG phage-like baseplate is recruited to the TssJLM membrane complex via multiple contacts and serves as assembly platform for tail tube/sheath polymerization. PLoS Genet.

[44] Park Y-J, Lacourse KD, Cambillau C, DiMaio F, Mougous JD (2018). Structure of the type VI secretion system TssK-TssF-TssG baseplate subcomplex revealed by cryo-electron microscopy. Nat Commun.

[45] Nazarov S, Schneider JP, Brackmann M, Goldie KN, Stahlberg H (2018). Cryo-EM reconstruction of Type VI secretion system baseplate and sheath distal end. EMBO J.

[46] Vanlioğlu E, Santin YG, Filella-Merce I, Pellarin R, Cascales E (2023). Coevolution-guided mapping of the type VI secretion membrane complex-baseplate interface. J Mol Biol.

[47] Nguyen VS, Logger L, Spinelli S, Legrand P, Huyen Pham TT (2017). Type VI secretion TssK baseplate protein exhibits structural similarity with phage receptor-binding proteins and evolved to bind the membrane complex. Nat Microbiol.

[48] English G, Byron O, Cianfanelli FR, Prescott AR, Coulthurst SJ (2014). Biochemical analysis of TssK, a core component of the bacterial Type VI secretion system, reveals distinct oligomeric states of TssK and identifies a TssK-TssFG subcomplex. Biochem J.

[49] Liebl D, Robert-Genthon M, Job V, Cogoni V, Attrée I (2019). Baseplate component TssK and spatio-temporal assembly of T6SS in *Pseudomonas aeruginosa*. Front Microbiol.

[50] Zoued A, Cassaro CJ, Durand E, Douzi B, España AP (2016). Structure-function analysis of the TssL cytoplasmic domain reveals a new interaction between the type VI secretion baseplate and membrane complexes. J Mol Biol.

[51] Douzi B, Logger L, Spinelli S, Blangy S, Cambillau C (2018). Structure-function analysis of the C-terminal domain of the type VI secretion TssB tail sheath subunit. J Mol Biol.

[52] Mougous JD, Gifford CA, Ramsdell TL, Mekalanos JJ (2007). Threonine phosphorylation post-translationally regulates protein secretion in *Pseudomonas aeruginosa*. Nat Cell Biol.

[53] Lin J-S, Pissaridou P, Wu H-H, Tsai M-D, Filloux A (2018). TagF-mediated repression of bacterial type VI secretion systems involves a direct interaction with the cytoplasmic protein Fha. J Biol Chem.

[54] Silverman JM, Austin LS, Hsu F, Hicks KG, Hood RD (2011). Separate inputs modulate phosphorylation-dependent and -independent type VI secretion activation. Mol Microbiol.

[55] Basler M, Ho BT, Mekalanos JJ (2013). Tit-for-tat: type VI secretion system counterattack during bacterial cell-cell interactions. Cell.

[56] Casabona MG, Silverman JM, Sall KM, Boyer F, Couté Y (2013). An ABC transporter and an outer membrane lipoprotein participate in posttranslational activation of type VI secretion in *Pseudomonas aeruginosa*. Environ Microbiol.

[57] Hsu F, Schwarz S, Mougous JD (2009). TagR promotes PpkA-catalysed type VI secretion activation in *Pseudomonas aeruginosa*. Mol Microbiol.

[58] Lin J-S, Wu H-H, Hsu P-H, Ma L-S, Pang Y-Y (2014). Fha interaction with phosphothreonine of TssL activates type VI secretion in *Agrobacterium tumefaciens*. PLoS Pathog.

[59] Ostrowski A, Cianfanelli FR, Porter M, Mariano G, Peltier J (2018). Killing with proficiency: integrated post-translational regulation of an offensive Type VI secretion system. PLoS Pathog.

[60] Ziveri J, Chhuon C, Jamet A, Rytter H, Prigent G (2019). Critical role of a sheath phosphorylation site on the assembly and function of an atypical type VI secretion system. Mol Cell Proteomics.

[61] Brunet YR, Hénin J, Celia H, Cascales E (2014). Type VI secretion and bacteriophage tail tubes share a common assembly pathway. EMBO Rep.

[62] Zoued A, Durand E, Santin YG, Journet L, Roussel A (2017). TssA: The cap protein of the Type VI secretion system tail. Bioessays.

[63] Pukatzki S, Ma AT, Revel AT, Sturtevant D, Mekalanos JJ (2007). Type VI secretion system translocates a phage tail spike-like protein into target cells where it cross-links actin. Proc Natl Acad Sci.

[64] Spínola-Amilibia M, Davó-Siguero I, Ruiz FM, Santillana E, Medrano FJ (2016). The structure of VgrG1 from *Pseudomonas aeruginosa*, the needle tip of the bacterial type VI secretion system. Acta Crystallogr D Struct Biol.

[65] Uchida K, Leiman PG, Arisaka F, Kanamaru S (2014). Structure and properties of the C-terminal β-helical domain of VgrG protein from *Escherichia coli* O157. J Biochem.

[66] Durand E, Cambillau C, Cascales E, Journet L (2014). VgrG, Tae, Tle, and beyond: the versatile arsenal of Type VI secretion effectors. Trends Microbiol.

[67] Alcoforado Diniz J, Liu YC, Coulthurst SJ (2015). Molecular weaponry: diverse effectors delivered by the Type VI secretion system. Cell Microbiol.

[68] Unterweger D, Kostiuk B, Pukatzki S (2017). Adaptor proteins of Type VI secretion system effectors. Trends Microbiol.

[69] Bondage DD, Lin JS, Ma LS, Kuo CH, Lai EM (2016). VgrG C terminus confers the type VI effector transport specificity and is required for binding with PAAR and adaptor–effector complex. Proc Natl Acad Sci.

[70] Shneider MM, Buth SA, Ho BT, Basler M, Mekalanos JJ (2013). PAAR-repeat proteins sharpen and diversify the type VI secretion system spike. Nature.

[71] Ballister ER, Lai AH, Zuckermann RN, Cheng Y, Mougous JD (2008). *In vitro* self-assembly of tailorable nanotubes from a simple protein building block. Proc Natl Acad Sci.

[72] Wang J, Brackmann M, Castaño-Díez D, Kudryashev M, Goldie KN (2017). Cryo-EM structure of the extended type VI secretion system sheath–tube complex. Nat Microbiol.

[73] Clemens DL, Ge P, Lee B-Y, Horwitz MA, Zhou ZH (2015). Atomic structure of T6SS reveals interlaced array essential to function. Cell.

[74] Salih O, He S, Planamente S, Stach L, MacDonald JT (2018). Atomic structure of type VI contractile sheath from *Pseudomonas aeruginosa*. Structure.

[75] Basler M (2015). Type VI secretion system: secretion by a contractile nanomachine. Phil Trans R Soc B.

[76] Chang Y-W, Rettberg LA, Ortega DR, Jensen GJ (2017). *In vivo* structures of an intact type VI secretion system revealed by electron cryotomography. EMBO Rep.

[77] Ge P, Scholl D, Leiman PG, Yu X, Miller JF (2015). Atomic structures of a bactericidal contractile nanotube in its pre- and postcontraction states. Nat Struct Mol Biol.

[78] Bönemann G, Pietrosiuk A, Diemand A, Zentgraf H, Mogk A (2009). Remodelling of VipA/VipB tubules by ClpV-mediated threading is crucial for type VI protein secretion. EMBO J.

[79] Schlieker C, Zentgraf H, Dersch P, Mogk A (2005). ClpV, a unique Hsp100/Clp member of pathogenic proteobacteria. Biol Chem.

[80] Pietrosiuk A, Lenherr ED, Falk S, Bönemann G, Kopp J (2011). Molecular basis for the unique role of the AAA+ chaperone ClpV in type VI protein secretion. J Biol Chem.

[81] Kube S, Kapitein N, Zimniak T, Herzog F, Mogk A (2014). Structure of the VipA/B type VI secretion complex suggests a contraction-state-specific recycling mechanism. Cell Rep.

[82] Förster A, Planamente S, Manoli E, Lossi NS, Freemont PS (2014). Coevolution of the ATPase ClpV, the sheath proteins TssB and TssC, and the accessory protein TagJ/HsiE1 distinguishes type VI secretion classes. J Biol Chem.

[83] Hachani A, Lossi NS, Hamilton A, Jones C, Bleves S (2011). Type VI secretion system in *Pseudomonas aeruginosa*: secretion and multimerization of VgrG proteins. J Biol Chem.

[84] Brodmann M, Dreier RF, Broz P, Basler M (2017). *Francisella* requires dynamic type VI secretion system and ClpB to deliver effectors for phagosomal escape. Nat Commun.

[85] Heisler DB, Kudryashova E, Grinevich DO, Suarez C, Winkelman JD (2015). ACTIN-DIRECTED TOXIN. ACD toxin-produced actin oligomers poison formin-controlled actin polymerization. Science.

[86] Sana TG, Flaugnatti N, Lugo KA, Lam LH, Jacobson A (2016). *Salmonella* Typhimurium utilizes a T6SS-mediated antibacterial weapon to establish in the host gut. Proc Natl Acad Sci.

[87] Allsopp LP, Bernal P, Nolan LM, Filloux A (2020). Causalities of war: the connection between type VI secretion system and microbiota. Cell Microbiol.

[88] Speare L, Jackson A, Septer AN (2022). Calcium promotes T6SS-mediated killing and aggregation between competing symbionts. Microbiol Spectr.

[89] Yang Y, Pan D, Tang Y, Li J, Zhu K (2022). H3-T6SS of *Pseudomonas aeruginosa* PA14 contributes to environmental adaptation via secretion of a biofilm-promoting effector. Stress Biol.

[90] Jiang F, Waterfield NR, Yang J, Yang G, Jin Q (2014). A *Pseudomonas aeruginosa* type VI secretion phospholipase D effector targets both prokaryotic and eukaryotic cells. Cell Host Microbe.

[91] Storey D, McNally A, Åstrand M, Sa-Pessoa Graca Santos J, Rodriguez-Escudero I (2020). *Klebsiella pneumoniae* type VI secretion system-mediated microbial competition is PhoPQ controlled and reactive oxygen species dependent. PLoS Pathog.

[92] Trunk K, Coulthurst SJ, Quinn J (2019). A new front in microbial warfare-delivery of antifungal effectors by the Type VI secretion system. J Fungi.

[93] Yang X, Clemens DL, Lee B-Y, Cui Y, Zhou ZH (2019). Atomic structure of the *Francisella* T6SS central spike reveals a unique α-Helical Lid and a putative cargo. Structure.

[94] Brodmann M, Schnider ST, Basler M, Monack D (2021). Type VI secretion system and its effectors PdpC, PdpD, and OpiA contribute to *Francisella* virulence in *Galleria mellonella* larvae. Infect Immun.

[95] Long ME, Lindemann SR, Rasmussen JA, Jones BD, Allen L-AH (2013). Disruption of *Francisella tularensis* Schu S4 *iglI*, *iglJ*, and *pdpC* genes results in attenuation for growth in human macrophages and *in vivo* virulence in mice and reveals a unique phenotype for *pdpC*. Infect Immun.

[96] Cantlay S, Kaftanic C, Horzempa J (2022). PdpC, a secreted effector protein of the type six secretion system, is required for erythrocyte invasion by *Francisella tularensis* LVS. Front Cell Infect Microbiol.

[97] Ren A, Jia M, Liu J, Zhou T, Wu L (2023). Acquisition of T6SS effector TseL contributes to the emerging of novel epidemic strains of *Pseudomonas aeruginosa*. Microbiol Spectr.

[98] Robinson LA, Collins ACZ, Murphy RA, Davies JC, Allsopp LP (2022). Diversity and prevalence of type VI secretion system effectors in clinical *Pseudomonas aeruginosa* isolates. Front Microbiol.

[99] Sá-Pessoa J, López-Montesino S, Przybyszewska K, Rodríguez-Escudero I, Marshall H (2023). A trans-kingdom T6SS effector induces the fragmentation of the mitochondrial network and activates innate immune receptor NLRX1 to promote infection. Nat Commun.

[100] Salinero-Lanzarote A, Pacheco-Moreno A, Domingo-Serrano L, Durán D, Ormeño-Orrillo E (2019). The Type VI secretion system of *Rhizobium etli* Mim1 has a positive effect in symbiosis. FEMS Microbiol Ecol.

[101] Bladergroen MR, Badelt K, Spaink HP (2003). Infection-blocking genes of a symbiotic *Rhizobium leguminosarum* strain that are involved in temperature-dependent protein secretion. Mol Plant Microbe Interact.

[102] Ma L-S, Hachani A, Lin J-S, Filloux A, Lai E-M (2014). *Agrobacterium tumefaciens* deploys a superfamily of type VI secretion DNase effectors as weapons for interbacterial competition in planta. Cell Host Microbe.

[103] Pissaridou P, Allsopp LP, Wettstadt S, Howard SA, Mavridou DAI (2018). The *Pseudomonas aeruginosa* T6SS-VgrG1b spike is topped by a PAAR protein eliciting DNA damage to bacterial competitors. Proc Natl Acad Sci.

[104] Russell AB, LeRoux M, Hathazi K, Agnello DM, Ishikawa T (2013). Diverse type VI secretion phospholipases are functionally plastic antibacterial effectors. Nature.

[105] Miyata ST, Unterweger D, Rudko SP, Pukatzki S (2013). Dual expression profile of type VI secretion system immunity genes protects pandemic *Vibrio cholerae*. PLoS Pathog.

[106] Nolan LM, Cain AK, Clamens T, Furniss RCD, Manoli E (2021). Identification of Tse8 as a Type VI secretion system toxin from *Pseudomonas aeruginosa* that targets the bacterial transamidosome to inhibit protein synthesis in prey cells. Nat Microbiol.

[107] Whitney JC, Quentin D, Sawai S, LeRoux M, Harding BN (2015). An interbacterial NAD(P)(+) glycohydrolase toxin requires elongation factor Tu for delivery to target cells. Cell.

[108] Ahmad S, Wang B, Walker MD, Tran H-KR, Stogios PJ (2019). An interbacterial toxin inhibits target cell growth by synthesizing (p)ppApp. Nature.

[109] Lin J, Zhang W, Cheng J, Yang X, Zhu K (2017). A *Pseudomonas* T6SS effector recruits PQS-containing outer membrane vesicles for iron acquisition. Nat Commun.

[110] de Moraes MH, Hsu F, Huang D, Bosch DE, Zeng J (2021). An interbacterial DNA deaminase toxin directly mutagenizes surviving target populations. Elife.

[111] González-Magaña A, Altuna J, Queralt-Martín M, Largo E, Velázquez C (2022). The *P. aeruginosa* effector Tse5 forms membrane pores disrupting the membrane potential of intoxicated bacteria. Commun Biol.

[112] Mok BY, de Moraes MH, Zeng J, Bosch DE, Kotrys AV (2020). A bacterial cytidine deaminase toxin enables CRISPR-free mitochondrial base editing. Nature.

[113] Russell AB, Singh P, Brittnacher M, Bui NK, Hood RD (2012). A widespread bacterial type VI secretion effector superfamily identified using a heuristic approach. Cell Host Microbe.

[114] Ting S-Y, Bosch DE, Mangiameli SM, Radey MC, Huang S (2018). Bifunctional immunity proteins protect bacteria against FtsZ-Targeting ADP-ribosylating toxins. Cell.

[115] Molina-Santiago C, Pearson JR, Navarro Y, Berlanga-Clavero MV, Caraballo-Rodriguez AM (2019). The extracellular matrix protects *Bacillus subtilis* colonies from *Pseudomonas* invasion and modulates plant co-colonization. Nat Commun.

[116] Le N-H, Pinedo V, Lopez J, Cava F, Feldman MF (2021). Killing of Gram-negative and Gram-positive bacteria by a bifunctional cell wall-targeting T6SS effector. Proc Natl Acad Sci.

[117] Pei T, Kan Y, Wang Z, Tang M, Li H (2022). Delivery of an Rhs‐family nuclease effector reveals direct penetration of the gram‐positive cell envelope by a type VI secretion system in *Acidovorax citrulli*. mLife.

[118] LaCourse KD, Peterson SB, Kulasekara HD, Radey MC, Kim J (2018). Conditional toxicity and synergy drive diversity among antibacterial effectors. Nat Microbiol.

[119] Li C, Zhu L, Wang D, Wei Z, Hao X (2022). T6SS secretes an LPS-binding effector to recruit OMVs for exploitative competition and horizontal gene transfer. ISME J.

[120] Garcia EC, Perault AI, Marlatt SA, Cotter PA (2016). Interbacterial signaling via *Burkholderia* contact-dependent growth inhibition system proteins. Proc Natl Acad Sci.

[121] Song L, Pan J, Yang Y, Zhang Z, Cui R (2021). Contact-independent killing mediated by a T6SS effector with intrinsic cell-entry properties. Nat Commun.

[122] Wang D, Zhu L, Zhen X, Yang D, Li C (2022). A secreted effector with a dual role as a toxin and as a transcriptional factor. Nat Commun.

[123] Nikolic N, Bergmiller T, Vandervelde A, Albanese TG, Gelens L (2018). Autoregulation of mazEF expression underlies growth heterogeneity in bacterial populations. Nucleic Acids Res.

[124] Russell AB, LeRoux M, Hathazi K, Agnello DM, Ishikawa T (2013). Diverse type VI secretion phospholipases are functionally plastic antibacterial effectors. Nature.

[125] Han Y, Wang T, Chen G, Pu Q, Liu Q (2019). A *Pseudomonas aeruginosa* type VI secretion system regulated by CueR facilitates copper acquisition. PLoS Pathog.

[126] Si M, Zhao C, Burkinshaw B, Zhang B, Wei D (2017). Manganese scavenging and oxidative stress response mediated by type VI secretion system in *Burkholderia thailandensis*. Proc Natl Acad Sci.

[127] Zhu L, Xu L, Wang C, Li C, Li M (2021). T6SS translocates a micropeptide to suppress STING-mediated innate immunity by sequestering manganese. Proc Natl Acad Sci.

[128] Lin RJ, Capage M, Hill CW (1984). A repetitive DNA sequence, rhs, responsible for duplications within the *Escherichia coli* K-12 chromosome. J Mol Biol.

[129] Jackson AP, Thomas GH, Parkhill J, Thomson NR (2009). Evolutionary diversification of an ancient gene family (rhs) through C-terminal displacement. BMC Genomics.

[130] Poole SJ, Diner EJ, Aoki SK, Braaten BA, t’Kint de Roodenbeke C (2011). Identification of functional toxin/immunity genes linked to contact-dependent growth inhibition (CDI) and rearrangement hotspot (Rhs) systems. PLoS Genet.

[131] Zhang D, de Souza RF, Anantharaman V, Iyer LM, Aravind L (2012). Polymorphic toxin systems: comprehensive characterization of trafficking modes, processing, mechanisms of action, immunity and ecology using comparative genomics. Biol Direct.

[132] Pei T-T, Li H, Liang X, Wang Z-H, Liu G (2020). Intramolecular chaperone-mediated secretion of an Rhs effector toxin by a type VI secretion system. Nat Commun.

[133] Cianfanelli FR, Alcoforado Diniz J, Guo M, De Cesare V, Trost M (2016). VgrG and PAAR proteins define distinct versions of a functional type VI secretion system. PLoS Pathog.

[134] Koskiniemi S, Lamoureux JG, Nikolakakis KC, t’Kint de Roodenbeke C, Kaplan MD (2013). Rhs proteins from diverse bacteria mediate intercellular competition. Proc Natl Acad Sci.

[135] Whitney JC, Beck CM, Goo YA, Russell AB, Harding BN (2014). Genetically distinct pathways guide effector export through the type VI secretion system. Mol Microbiol.

[136] Hachani A, Allsopp LP, Oduko Y, Filloux A (2014). The VgrG proteins are “à la carte” delivery systems for bacterial type VI effectors. J Biol Chem.

[137] Alcoforado Diniz J, Coulthurst SJ (2015). Intraspecies competition in *Serratia marcescens* is mediated by type VI-secreted Rhs effectors and a conserved effector-associated accessory protein. J Bacteriol.

[138] Busby JN, Panjikar S, Landsberg MJ, Hurst MRH, Lott JS (2013). The BC component of ABC toxins is an RHS-repeat-containing protein encapsulation device. Nature.

[139] Günther P, Quentin D, Ahmad S, Sachar K, Gatsogiannis C (2022). Structure of a bacterial Rhs effector exported by the type VI secretion system. PLoS Pathog.

[140] Jurėnas D, Rosa LT, Rey M, Chamot-Rooke J, Fronzes R (2021). Mounting, structure and autocleavage of a type VI secretion-associated Rhs polymorphic toxin. Nat Commun.

[141] Tang L, Dong S, Rasheed N, Wu HW, Zhou N (2022). *Vibrio parahaemolyticus* prey targeting requires autoproteolysis-triggered dimerization of the type VI secretion system effector RhsP. Cell Reports.

[142] Silverman JM, Agnello DM, Zheng H, Andrews BT, Li M (2013). Haemolysin coregulated protein is an exported receptor and chaperone of type VI secretion substrates. Mol Cell.

[143] Ma J, Pan Z, Huang J, Sun M, Lu C (2017). The Hcp proteins fused with diverse extended-toxin domains represent a novel pattern of antibacterial effectors in type VI secretion systems. Virulence.

[144] Liang X, Moore R, Wilton M, Wong MJQ, Lam L (2015). Identification of divergent type VI secretion effectors using a conserved chaperone domain. Proc Natl Acad Sci.

[145] Unterweger D, Kostiuk B, Ötjengerdes R, Wilton A, Diaz-Satizabal L (2015). Chimeric adaptor proteins translocate diverse type VI secretion system effectors in *Vibrio cholerae*. EMBO J.

[146] Burkinshaw BJ, Liang X, Wong M, Le ANH, Lam L (2018). A type VI secretion system effector delivery mechanism dependent on PAAR and A chaperone-co-chaperone complex. Nat Microbiol.

[147] Liu Y, Zhang Z, Wang F, Li D-D, Li Y-Z (2020). Identification of type VI secretion system toxic effectors using adaptors as markers. Comput Struct Biotechnol J.

[148] Filloux A (2022). Bacterial protein secretion systems: game of types. Microbiology.

[149] Dar Y, Jana B, Bosis E, Salomon D (2022). A binary effector module secreted by a type VI secretion system. EMBO Rep.

[150] Flaugnatti N, Le TTH, Canaan S, Aschtgen M-S, Nguyen VS (2016). A phospholipase A1 antibacterial Type VI secretion effector interacts directly with the C-terminal domain of the VgrG spike protein for delivery. Mol Microbiol.

[151] Flaugnatti N, Rapisarda C, Rey M, Beauvois SG, Nguyen VA (2020). Structural basis for loading and inhibition of a bacterial T6SS phospholipase effector by the VgrG spike. EMBO J.

[152] Liang X, Kamal F, Pei T-T, Xu P, Mekalanos JJ (2019). An onboard checking mechanism ensures effector delivery of the type VI secretion system in *Vibrio cholerae*. Proc Natl Acad Sci.

[153] Wu C, Lien Y, Bondage D, Lin J, Pilhofer M (2020). Effector loading onto the VgrG carrier activates type VI secretion system assembly. EMBO Reports.

[154] Tchelet D, Keppel K, Bosis E, Salomon D (2023). *Vibrio parahaemolyticus* T6SS2 effector repertoires. Gut Microbes.

[155] Lopez J, Ly PM, Feldman MF, Stephen Trent M (2020). The tip of the VgrG spike is essential to functional type VI secretion system assembly in *Acinetobacter baumannii*. mBio.

[156] Ruhe ZC, Low DA, Hayes CS (2020). Polymorphic toxins and their immunity proteins: diversity, evolution, and mechanisms of delivery. Annu Rev Microbiol.

[157] Speare L, Woo M, Dunn AK, Septer AN, Dubilier N (2022). A putative lipoprotein mediates cell-cell contact for type VI secretion system-dependent killing of specific competitors. mBio.

[158] Ting S-Y, Martínez-García E, Huang S, Bertolli SK, Kelly KA (2020). Targeted depletion of bacteria from mixed populations by programmable adhesion with antagonistic competitor cells. Cell Host Microbe.

[159] Wettstadt S, Filloux A (2020). Manipulating the type VI secretion system spike to shuttle passenger proteins. PLoS One.

[160] Hood RD, Singh P, Hsu F, Güvener T, Carl MA (2010). A type VI secretion system of *Pseudomonas aeruginosa* targets a toxin to bacteria. Cell Host Microbe.

[161] Durán D, Bernal P, Vazquez-Arias D, Blanco-Romero E, Garrido-Sanz D (2021). *Pseudomonas fluorescens* F113 type VI secretion systems mediate bacterial killing and adaption to the rhizosphere microbiome. Sci Rep.

[162] Fridman CM, Keppel K, Gerlic M, Bosis E, Salomon D (2020). A comparative genomics methodology reveals a widespread family of membrane-disrupting T6SS effectors. Nat Commun.

[163] Jana B, Keppel K, Fridman CM, Bosis E, Salomon D (2022). Multiple T6SSs, mobile auxiliary modules, and effectors revealed in a systematic analysis of the *Vibrio parahaemolyticus* pan-genome. mSystems.

[164] Geller AM, Zlotkin D, Levy A (2021). Large-scale discovery of candidate type VI secretion effectors with antibacterial activity. Microbiology.

[165] Unterweger D, Miyata ST, Bachmann V, Brooks TM, Mullins T (2014). The *Vibrio cholerae* type VI secretion system employs diverse effector modules for intraspecific competition. Nat Commun.

[166] Rigard M, Bröms JE, Mosnier A, Hologne M, Martin A (2016). *Francisella tularensis* IglG belongs to a novel family of PAAR-like T6SS proteins and harbors a unique N-terminal extension required for virulence. PLoS Pathog.

[167] Salomon D, Kinch LN, Trudgian DC, Guo X, Klimko JA (2014). Marker for type VI secretion system effectors. Proc Natl Acad Sci.

[168] Jana B, Fridman CM, Bosis E, Salomon D (2019). A modular effector with a DNase domain and a marker for T6SS substrates. Nat Commun.

[169] Kanarek K, Fridman CM, Bosis E, Salomon D (2022). A new class of polymorphic T6SS effectors and tethers. Microbiology.

[170] Allsopp LP, Collins ACZ, Hawkins E, Wood TE, Filloux A (2022). RpoN/Sfa2-dependent activation of the *Pseudomonas aeruginosa* H2-T6SS and its cognate arsenal of antibacterial toxins. Nucleic Acids Res.

[171] Vazquez-Lopez J, Navarro-Garcia F (2020). In silico analyses of core proteins and putative effector and immunity proteins for T6SS in enterohemorrhagic *E. coli*. Front Cell Infect Microbiol.

[172] Habich A, Galeev A, Vargas VC, Vogler O, Ghoul M Core and accessory effectors of type VI secretion systems contribute differently to the intraspecific diversity of *Pseudomonas aeruginosa*. Microbiology.

[173] Bingle LE, Bailey CM, Pallen MJ (2008). Type VI secretion: a beginner’s guide. Curr Opin Microbiol.

[174] Gallique M, Decoin V, Barbey C, Rosay T, Feuilloley MGJ (2017). Contribution of the *Pseudomonas fluorescens* MFE01 Type VI secretion system to biofilm formation. PLoS One.

[175] Decoin V, Barbey C, Bergeau D, Latour X, Feuilloley MGJ (2014). A type VI secretion system is involved in *Pseudomonas fluorescens* bacterial competition. PLoS One.

[176] Borrero de Acuña JM, Bernal P (2021). Plant holobiont interactions mediated by the type VI secretion system and the membrane vesicles: promising tools for a greener agriculture. Environ Microbiol.

[177] Bernal P, Civantos C, Pacheco-Sánchez D, Quesada JM, Filloux A (2023). Transcriptional organization and regulation of the *Pseudomonas putida* K1 type VI secretion system gene cluster. Microbiology.

[178] Bayer-Santos E, Lima LDP, Ceseti L de M, Ratagami CY, de Santana ES (2018). *Xanthomonas citri* T6SS mediates resistance to *Dictyostelium* predation and is regulated by an ECF σ factor and cognate Ser/Thr kinase. Environ Microbiol.

[179] Bernal P, Murillo-Torres M, Allsopp LP (2020). Integrating signals to drive type VI secretion system killing. Environ Microbiol.

[180] Jaskólska M, Stutzmann S, Stoudmann C, Blokesch M (2018). QstR-dependent regulation of natural competence and type VI secretion in *Vibrio cholerae*. Nucleic Acids Res.

[181] Alves JA, Leal FC, Previato-Mello M, da Silva Neto JF (2022). A quorum sensing-regulated type VI secretion system containing multiple nonredundant VgrG proteins is required for interbacterial competition in *Chromobacterium violaceum*. Microbiol Spectr.

[182] Allsopp LP, Wood TE, Howard SA, Maggiorelli F, Nolan LM (2017). RsmA and AmrZ orchestrate the assembly of all three type VI secretion systems in *Pseudomonas aeruginosa*. Proc Natl Acad Sci.

[183] Lin L, Lezan E, Schmidt A, Basler M (2019). Abundance of bacterial Type VI secretion system components measured by targeted proteomics. Nat Commun.

[184] Deng W, Marshall NC, Rowland JL, McCoy JM, Worrall LJ (2017). Assembly, structure, function and regulation of type III secretion systems. Nat Rev Microbiol.

[185] Wanford JJ, Hachani A, Odendall C (2022). Reprogramming of cell death pathways by bacterial effectors as a widespread virulence strategy. Infect Immun.

